# Alterations in the p53 isoform ratio govern breast cancer cell fate in response to DNA damage

**DOI:** 10.1038/s41419-022-05349-9

**Published:** 2022-10-28

**Authors:** Luiza Steffens Reinhardt, Xiajie Zhang, Kira Groen, Brianna C. Morten, Geoffry N. De Iuliis, Antony W. Braithwaite, Jean-Christophe Bourdon, Kelly A. Avery-Kiejda

**Affiliations:** 1 School of Biomedical Sciences and Pharmacy, College of Health, Medicine and Wellbeing, The University of Newcastle, Callaghan, NSW Australia; 2 Hunter Medical Research Institute, New Lambton Heights, NSW Australia; 3 Priority Research Centre for Reproductive Science, School of Environmental and Life Sciences, College of Engineering, Science and Environment, The University of Newcastle, Callaghan, NSW Australia; 4 Children’s Medical Research Institute, The University of Sydney, Sydney, NSW Australia; 5 Department of Pathology, School of Medicine, The University of Otago, Dunedin, New Zealand; 6 Dundee Cancer Centre, Ninewells Hospital and Medical School, The University of Dundee, Dundee, UK

**Keywords:** Breast cancer, DNA damage and repair, Apoptosis

## Abstract

Our previous studies have shown that p53 isoform expression is altered in breast cancer and related to prognosis. In particular, a high ∆40p53:p53α ratio is associated with worse disease-free survival. In this manuscript, the influence of altered Δ40p53 and p53α levels on the response to standard of care DNA-damaging agents used in breast cancer treatment was investigated in vitro. Our results revealed that a high Δ40p53:p53α ratio causes cells to respond differently to doxorubicin and cisplatin treatments. Δ40p53 overexpression significantly impairs the cells’ sensitivity to doxorubicin through reducing apoptosis and DNA damage, whereas Δ40p53 knockdown has the opposite effect. Further, a high Δ40p53:p53α ratio inhibited the differential expression of several genes following doxorubicin and promoted DNA repair, impairing the cells’ canonical response. Overall, our results suggest that the response of breast cancer cells to standard of care DNA-damaging therapies is dependent on the expression of p53 isoforms, which may contribute to outcomes in breast cancer.

## Introduction

The *TP53* gene, known as the “guardian of the genome”, is one of the most commonly mutated genes in cancer, alongside the proto-oncogene *PI3KCA* [1]. Somatic mutation of the tumour suppressor gene *TP53* is related to breast cancer subtype, tumour progression, resistance to therapy, and poor prognosis in breast cancer; however, its overall mutation frequency of ~25% is less than expected for a protein that executes key functions that maintain genome integrity [2, 3].

DNA-damaging agents such as cyclophosphamide, cisplatin (CIS), and doxorubicin (DOX) are frequently used to treat breast cancer [4], but the mechanisms of resistance to these agents are poorly defined. The p53 protein is activated by a range of cellular stressors such as DNA-damaging agents, hypoxia, and nutrient starvation. This triggers multiple signalling pathways involved in the DNA damage response (DDR), including cell-cycle arrest, apoptosis, and DNA repair [5–7]. Full-length p53 (referred to herein as p53α) is expressed at low levels and maintained in a steady state by human double minute 2 (HDM2) and its regulators p14^ARF^ and p16^INK4A^ [8–10]. Upon DNA damage, p53α is activated through post-translational modifications (PTMs) including phosphorylation of serine residues within the transactivation domain 1 (TAD1) [9, 11]. In its active conformation, p53α forms a tetramer and binds to p53 responsive elements (REs) in the promoter region of target genes to enhance or inhibit their expression. Interestingly, p53α promotes some DNA repair pathways, including the base excision repair, mismatch repair, and nucleotide excision repair pathways [12–14], while inhibiting DNA double-strand break (DSB) repair pathways, including the homologous recombination (HR), non-homologous end-joining, and single-stranded annealing pathways [11, 15–25].

Given the importance of p53α in tumour suppression [2], and the low mutation frequency in sporadic hormone-dependent breast cancer, other mechanisms may be responsible for the disruption of this critical tumour suppressor. *TP53* is expressed as p53α as well as 12 smaller isoforms that can modulate its function: p53β, p53γ, p53Ψ, Δ133p53, Δ133p53β, Δ133p53γ, Δ40p53, Δ40p53β, Δ40p53γ, Δ160p53, Δ160p53β, and Δ160p53γ [26–28]. Studies indicate that the isoforms can enhance or inhibit the ability of p53α to transactivate certain target genes and to induce apoptosis [5, 26, 29–31], senescence [31–34], and angiogenesis [32, 35, 36]. The p53 isoforms can be generated through alternative splicing (Δ40, β, γ), alternative promoter usage (Δ133, Δ160), and alternative initiation of translation (Δ40, Δ160) [26, 27]. All N-terminal isoforms lack the HDM2 binding domain and this is thought to contribute to their increased stability [37].

Our previous studies have shown that Δ40p53 is the most highly expressed p53 isoform in breast cancer, aside from p53α itself, with significantly higher expression in tumour samples compared to matched normal adjacent tissue [38]. Additionally, a high Δ40p53:p53α ratio is associated with increased likelihood of metastasis and recurrence, suggesting that this isoform plays a role in breast cancer carcinogenesis and that it may alter therapeutic outcomes [38, 39].

Δ40p53 is known to play a key role in the response to endoplasmic reticulum stress [33], however, little is known about the underlying mechanisms by which Δ40p53 orchestrates an altered p53 response to DNA damage, especially in regard to p53 pathways important in carcinogenesis, such as DNA repair and apoptosis. Most chemotherapies used to treat breast cancer work by inflicting DNA-damage to drive cancer cells towards apoptosis. Therefore, defining the role of Δ40p53 in the p53-mediated DDR may uncover novel chemoresistance mechanisms. In this study, the role of Δ40p53 in the p53-mediated DDR to DOX and CIS was investigated. Our data show that altered levels of Δ40p53 modulate cell fate in a drug-dependent fashion and that Δ40p53 is involved in modulating the p53-mediated DDR by promoting DNA repair and antagonising apoptosis, in response to chemotherapy-driven DNA-damage, which may result in decreased sensitivity to these therapies.

## Results

### p53α and Δ40p53 are highly expressed in response to DNA damage

**Fig. 1:**
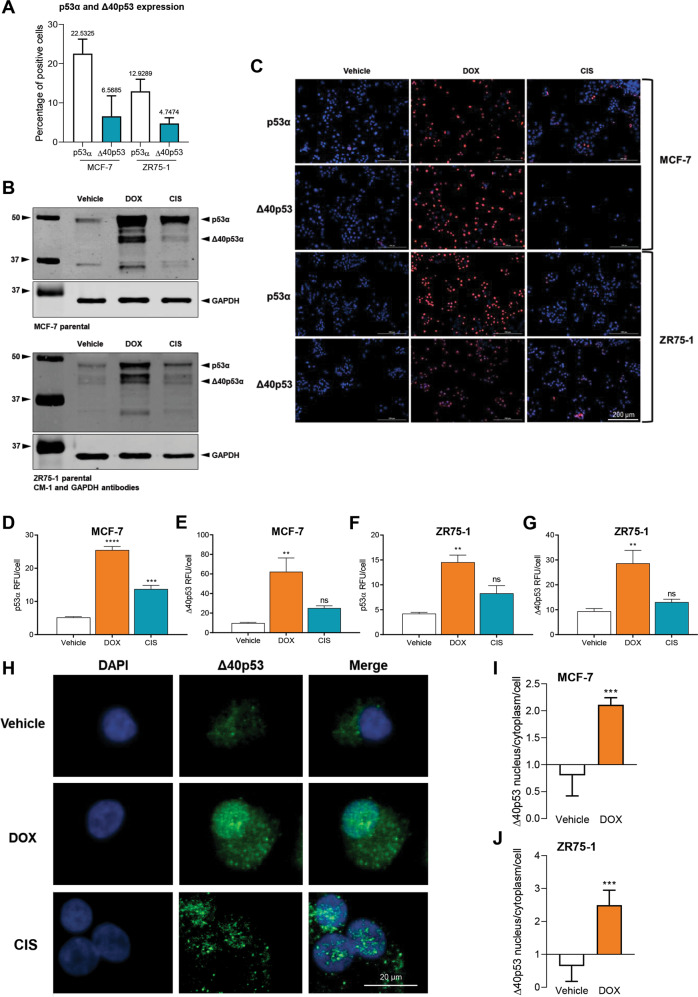
p53α and Δ40p53 are highly expressed in response to DNA damage in MCF-7 and ZR75-1 cell lines. **A** The percentage of cells positive for p53α and Δ40p53 at the basal level in MCF-7 and ZR75-1 cells analysed by immunofluorescence using DO-1 (1:100) and KJC40 (1:70) primary antibodies. The corresponding average percentage is shown at the top of the columns. Data shown represent three independent experiments of three technical replicates. **B** Representative immunoblotting analysis of MCF-7 and ZR75-1 cell extracts (40 µg) treated with vehicle (water), DOX or CIS (24 h). CM-1 (1 μg/ml) and GAPDH (1 μg/ml; loading control) primary antibodies were used. Data shown represent three independent experiments. Fold-change expression relative to vehicle-treated cells for MCF-7 cell extracts: p53α 20.9-fold (DOX) and 5.5-fold (CIS), Δ40p53 43.6-fold (DOX) and 13.3-fold (CIS); and for ZR75-1 cell extracts: p53α 8.9-fold (DOX) and 7.7-fold (CIS), Δ40p53 9-fold (DOX) and 3-fold (CIS). **C** Immunofluorescence images of p53α and Δ40p53 staining before and after treatment with vehicle (water), DOX or CIS (24 h) in MCF-7 and ZR75-1 cell lines. DO-1 (1:100) and KJC40 (1:70) primary antibodies were used and cell nuclei were stained with DAPI. Data shown represent three independent experiments. For negative controls of primary and secondary antibodies see Supplementary Fig. 1. **D** p53α and **E** Δ40p53 expression in relative fluorescence units (RFU) normalised to cell count after DOX or CIS treatment (24 h) in the MCF-7 cell line. Data shown represent three independent experiments of three technical replicates. **F** p53α and **G** Δ40p53 expression after DOX or CIS treatment (24 h) in the ZR75-1 cell line. Data shown represent three independent experiments of three technical replicates. **H** The nuclear/cytoplasmic ratio expression of Δ40p53 after treatment with vehicle or DOX (24 h) in MCF-7 cells and **I** ZR75-1 cells. Data shown represent three independent experiments of three technical replicates. **J** Immunofluorescence images of Δ40p53 staining after treatment with vehicle or DOX (24 h) in ZR75-1 cells. KJC40 (1:70) primary antibody was used and cell nuclei are stained with DAPI. Results are shown as the mean ± SD. Statistical analyses were carried out using one-way ANOVA followed by Dunnett’s post-test **(D–G)** or unpaired *t*-test **(H, I)**. Results were considered significant at *p* < 0.05; **p < 0.01, ***p < 0.001, ****p < 0.0001.

To determine if Δ40p53 levels could be modulated by DNA-damaging chemotherapies, Δ40p53 expression was analysed following 24 h treatment with DOX or CIS. For this analysis, two breast cancer cell lines that harbour WTp53 were used, MCF-7 and ZR75-1. Following treatment, protein was extracted from cell pellets or cells were fixed for immunofluorescence of p53α and Δ40p53. At the basal level, in MCF-7 cells, approximately 22% and 6% of cells stained for p53α and Δ40p53, respectively, whereas, in ZR75-1 cells, approximately 12% and 4% of cells stained for p53α and Δ40p53, respectively (Fig. 1A). In both cell lines, p53α (Fig. 1B, D, F) and Δ40p53 (Fig. 1B, C, E, G) were highly expressed after DOX or CIS treatment (Fig. 1B–D). In the absence of treatment, endogenously expressed Δ40p53 was localised predominantly in the cytoplasm, confirming previous in vitro studies overexpressing this isoform [40, 41] (Fig. 1H). However, after DOX treatment, Δ40p53’s nuclear expression increased by 2-fold in MCF-7 cells (Fig. 1I) and by more than 2.5-fold in ZR75-1 cells (Fig. 1J).

### Knockdown of Δ40p53 alters cell cycle progression after doxorubicin treatment

Having demonstrated that Δ40p53 is induced by genotoxic agents, we next explored the function of Δ40p53 in the DDR by using breast cancer cell lines in which Δ40p53 has been stably knocked down (shΔ40p53) or overexpressed (Δ40p53) [42]. To compare these results with the function of p53α, a TAp53 knockdown subline was used along with sublines containing a non-targeting control shRNA (shNT) or empty vector (LeGO) [42]. The response of these sublines to DOX and CIS was examined by cell cycle analysis.

**Fig. 2:**
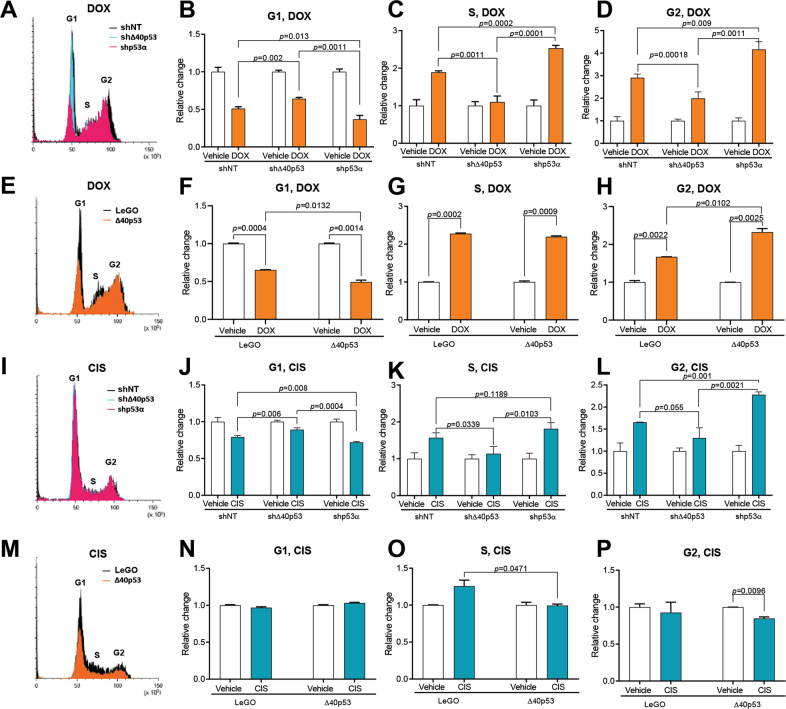
Modulation of Δ40p53 levels alters cell cycle progression in response to DNA damage. **A** Representative histograms of DOX-treated (24 h) MCF-7-shNT (shown in black), MCF-7-shΔ40p53 (shown in blue) and MCF-7-shp53α (shown in pink) sublines. Relative change in **B** G1-phase, **C** S-phase and **D** G2-phase in MCF-7-shNT, MCF-7-shΔ40p53 and MCF-7-shp53α sublines. **E** Representative histograms of DOX-treated (24 h) MCF-7-LeGO (shown in black) and MCF-7-Δ40p53 (shown in orange) sublines. Relative change in **F** G1-phase, **G** S-phase and **H** G2-phase in MCF-7-LeGO and MCF-7-Δ40p53 sublines. **I** Representative histograms of CIS-treated (24 h) MCF-7-shNT (shown in black), MCF-7-shΔ40p53 (shown in blue) and MCF-7-shp53α (shown in pink) sublines. Relative change in **J** G1-phase, **K** S-phase and **L** G2-phase in MCF-7-shNT, MCF-7-shΔ40p53 and MCF-7-shp53α sublines. **M** Representative histograms of CIS-treated (24 h) MCF-7-LeGO (shown in black) and MCF-7-Δ40p53 (shown in orange) sublines. Relative change in **N** G1-phase, **O** S-phase and **P** G2-phase in MCF-7-LeGO and MCF-7-Δ40p53 sublines. Data shown represent three independent experiments of three technical replicates. Results are shown as the mean ± SD Statistical analyses were carried out using an unpaired t-test. Results were considered significant at *p* < 0.05.

Knockdown of Δ40p53 in MCF-7 cells led to a significant increase in the G1 population (*p* = 0.002) and a decrease in the S (*p* = 0.0011) and G2 populations (*p* = 0.00018) after DOX treatment compared to shNT cells (Fig. 2A–D), whereas in the ZR75-1 cells, Δ40p53-knockdown had limited impact on G1 phase but led to a significantly reduced induction of G2 in response to either drug (DOX-treated cells: *p* = 0.048; CIS-treated cells: *p* = 0.002), when compared to shNT transduced cells (Supplementary Fig. 2A–F). Knockdown of p53α significantly reduced the number of cells in G1 and increased the proportion of cells in G2 in both MCF-7 and ZR75-1 cells following CIS or DOX treatment compared to shNT cells (*p* < 0.05 for all comparisons, except G2 of CIS-treated ZR75-1-shp53α vs. shNT, see Figure for details; Fig. 2A–D, I–L, Supplementary Fig. 2A–F), and shΔ40p53 cells (*p* < 0.05 for all comparisons, Fig. 2A–D, I–L, Supplementary Fig. 2A–F). In the same manner, the MCF-7-Δ40p53 subline exhibited a significant reduction in the proportion of cells in G1 (*p* = 0.0132) and a significant increase in the proportion of cells in G2 (*p* = 0.0102) in response to DOX when compared to MCF-7-LeGO cells (Fig. 2E–H). However, the MCF-7-Δ40p53 subline had a lower proportion of cells in G2 in response to CIS when compared to vehicle-treated cells (*p* = 0.0096) (Fig. 2M, P).

Overall, DOX led to more pronounced cell cycle changes when compared to CIS, with a high Δ40p53:p53α ratio (Δ40p53-overexpression or p53α-knockdown) [42] reducing the number of cells in G1 and enhancing the proportion of cells in G2 following DOX; while the opposite was observed in cells with a low Δ40p53:p53α ratio (Δ40p53-knockdown) (Fig. 2, Supplementary Fig. 2A–C).

### Knockdown of Δ40p53 enhances apoptosis after doxorubicin treatment

DNA damage can trigger cell cycle arrest, leading to DNA repair, or cell death. Our results have shown that the Δ40p53:p53α ratio can alter cell cycle progression at specific phases depending on the DNA-damaging agent, but whether the cells repaired the damaged DNA and continued to survive or underwent apoptosis remains unknown. Therefore, we investigated whether an alteration in Δ40p53 or p53α expression affected apoptosis induced by DOX or CIS.

**Fig. 3:**
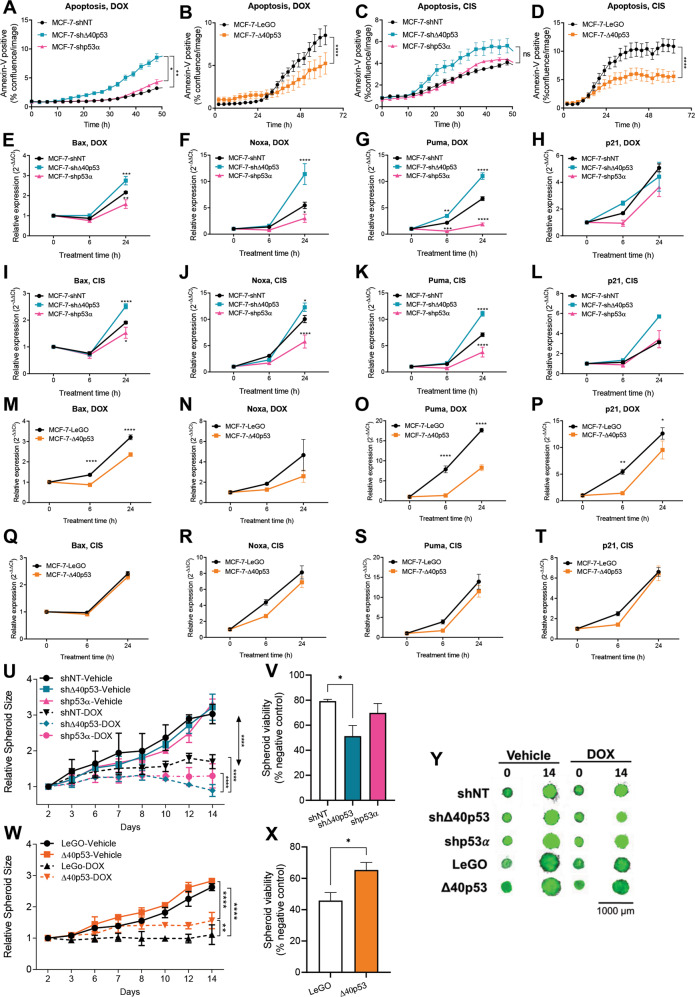
Knockdown of Δ40p53 enhances apoptosis after DOX treatment, whereas Δ40p53 overexpression decreases apoptosis following DOX or CIS treatment. Annexin-V positive cells normalised to confluence in **A** MCF-7-shNT, MCF-7-shΔ40p53 and MCF-7-shp53α sublines and **B** MCF-7-LeGO and MCF-7-Δ40p53 sublines following DOX treatment. Data shown represent four independent experiments of three technical replicates. Annexin-V positive cells normalised to confluence in **C** MCF-7-shNT, MCF-7-shΔ40p53 and MCF-7-shp53α sublines and **D** MCF-7-LeGO and MCF-7-Δ40p53 sublines following CIS treatment. Data shown represent four independent experiments of three technical replicates. mRNA levels of **E** *BAX*, **F** *NOXA*, **G** *PUMA* and **H** *CDKN1A* in response to DOX normalised to vehicle-treated cells in MCF-7-shNT, MCF-7-shΔ40p53 and MCF-7-shp53α sublines. Data shown represent three independent experiments of three technical replicates. mRNA levels of **I** *BAX*, **J** *NOXA*, **K** *PUMA* and **L** *CDKN1A* in response to CIS normalised to vehicle-treated cells in MCF-7-shNT, MCF-7-shΔ40p53 and MCF-7-shp53α sublines. Data shown represent three independent experiments of three technical replicates. mRNA levels of **M** *BAX*, **N** *NOXA*, **O** *PUMA* and **P** *CDKN1A* in response to DOX normalised to vehicle-treated cells in MCF-7-LeGO and MCF-7-Δ40p53 sublines. Data shown represent three independent experiments of three technical replicates. mRNA levels of **Q** *BAX*, **R** *NOXA*, **S** *PUMA* and **T** *CDKN1A* in response to CIS normalised to vehicle-treated cells in MCF-7-LeGO and MCF-7-Δ40p53 sublines. Data shown represent three independent experiments of three technical replicates. **U** Spheroid size normalised to size prior treatment with DOX and **V** spheroid viability normalised to vehicle-treated spheroids in MCF-7-shNT, MCF-7-shΔ40p53 and MCF-7-shp53α sublines. **W** Spheroid size normalised to size prior to treatment with DOX and **X** spheroid viability normalised to vehicle-treated spheroids in MCF-7-LeGO and MCF-7-Δ40p53 sublines. Data shown represent three independent experiments of four technical replicates. **Y** Representative images of vehicle or DOX-treated MCF-7 sublines spheroids on day 0 (prior treatment) and day 14. Results are shown as the mean ± SD. Statistical analyses were carried out using two-way ANOVA followed by Sidak’s post-test **(A**–**U, W)**, using one-way ANOVA followed by Tukey’s post-test **V** or unpaired *t*-test **X**. Results were considered significant at *p* < 0.05; *p < 0.05, **p < 0.01, ***p < 0.001, ****p < 0.0001.

In MCF-7 sublines, Δ40p53 knockdown increased the rate of apoptosis and Δ40p53 overexpression attenuated apoptosis in response to DOX (Fig. 3A, B). Knockdown of p53α had limited effects on the apoptosis rate when compared to shNT cells (Fig. 3A). The MCF-7-Δ40p53 subline exhibited a decreased apoptosis rate compared to LeGO cells when treated with CIS, however, there were no differences in the apoptosis rate in the knockdown sublines (Fig. 3C, D). In ZR75-1 sublines, knockdown of Δ40p53 resulted in a significant reduction in DOX-induced cell viability after 96 h of treatment (*p* = 0.0363) and apoptosis after 72 h of treatment (*p* < 0.0001), whereas the subline in which p53α was knocked down demonstrated less sensitivity to the treatment (Supplementary Fig. 2G, H), but no differences were found in CIS-treated cells (Supplementary Fig. 2I). These results show that in response to two DNA-damaging agents, high Δ40p53 levels inhibited apoptosis.

Next, we investigated p53-dependent cell cycle and apoptosis-related gene expression by RT-qPCR. We looked at the relative mRNA expression changes of four commonly known p53-dependent target genes including *CDKN1A* (p21) and three pro-apoptotic markers *BAX*, *PUMA*, and *NOXA* (Fig. 3E–T, Supplementary Fig. 2J–L). In the MCF-7 cells, all genes were upregulated 24 h after treatment with either DNA-damaging agent. The expression level of all three pro-apoptotic genes was significantly upregulated (*p* < 0.05) when Δ40p53 was knocked down and significantly downregulated (*p* < 0.05) when p53α was knocked down (Fig. 3E–G, I–K). The expression of *CDKN1A* was not significantly different among the knockdown MCF-7 sublines in response to DOX or CIS treatment (Fig. 3H, L). This supports our results that Δ40p53 knockdown led to increased apoptosis. In contrast, the induction of *BAX*, *NOXA*, *PUMA*, and *CDKN1A* expression was inhibited in MCF-7-Δ40p53 cells following DOX treatment (Fig. 3M–P). However, Δ40p53 overexpression had no effect on CIS-mediated induction of these genes (Fig. 3Q–T). In ZR75-1 sublines, no statistically significant differences were observed in the expression of *BAX*, *PUMA*, and *NOXA* after DOX treatment (Supplementary Fig. 2J–L).

To confirm the differential sensitivity to DOX among the cell sublines, cell spheroids were generated and their size and viability were evaluated (Fig. 3U–Y, Supplementary Fig. 2M, N). Δ40p53 knockdown decreased spheroid size and viability following treatment in both MCF-7 and ZR75-1 cells (Fig. 3U, V, Supplementary Fig. 2M, N), whereas, MCF-7-Δ40p53 spheroids showed less sensitivity to DOX when compared to LeGO spheroids (Fig. 3W, X).

These results suggest that varying Δ40p53:p53α ratios promote different cellular decisions by modulating the transactivation of p53α-target genes in a DNA damaging agent-specific fashion, resulting in differential apoptosis rates and sensitivity to DOX.

### p53α is upregulated by Δ40p53 knockdown after doxorubicin treatment

**Fig. 4:**
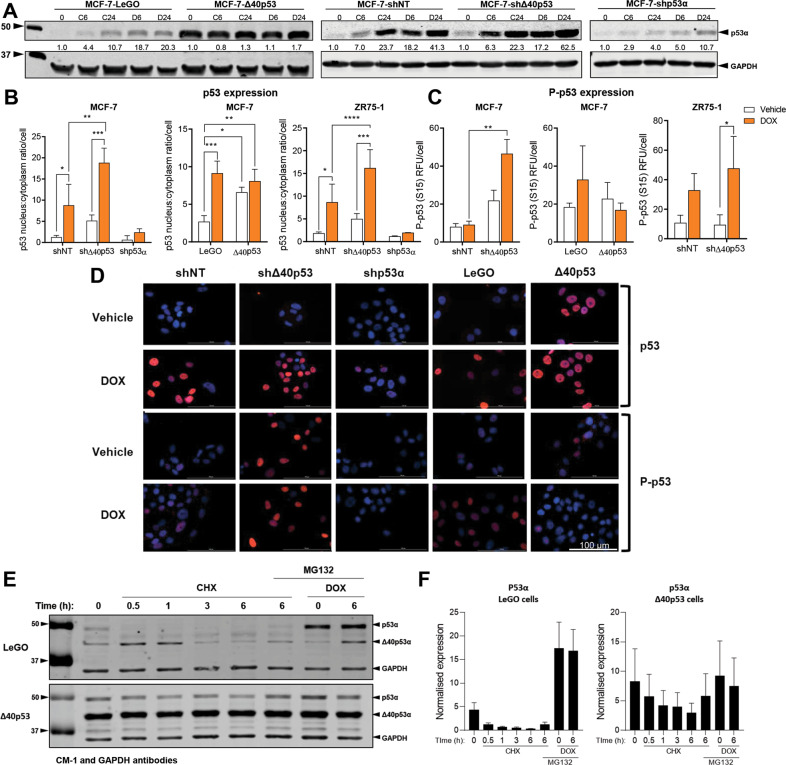
Δ40p53 knockdown increases the expression and phosphorylation of p53 following DOX treatment. **A** Representative immunoblotting analysis from 40 µg of protein extracts showed that p53 protein levels were upregulated after CIS and DOX treatment (**C**, **D** represent CIS and DOX respectively and digits indicate time (hours) after treatment) in the MCF-7-shNT, MCF-7-shΔ40p53, MCF-7-shp53α and MCF-7-LeGO sublines, but not in the MCF-7-Δ40p53 subline. Fold-change expression is indicated by the digits between p53 and GAPDH western blots. CM-1 (1 μg/ml) and GAPDH (1 μg/ml; loading control) primary antibodies were used. Data shown represent three independent experiments. **B** p53α nucleus/cytoplasmic ratio and **C** P-p53 (S15) expression measured by immunofluorescence after DOX treatment (24 h) in MCF-7 (-shNT, -shΔ40p53, -shp53α, LeGO and Δ40p53) and ZR75-1 (-shNT, -shΔ40p53, -shp53α) sublines. Data shown represent three independent experiments of three technical replicates. **D** Immunofluorescence images of p53α staining after treatment with vehicle (water) or DOX (24 h) in MCF-7 (-shNT, -shΔ40p53, -shp53α, LeGO and Δ40p53) sublines. DO-1 (1:100) and P-p53 (S15) (1:400) primary antibodies were used and cell nuclei were stained with DAPI. **E** Representative immunoblotting analysis of MCF-7-LeGO and MCF-7- Δ40p53 cell extracts treated with vehicle (water), CHX (40 µg/mL), MG132 (10 µM) and/or DOX (1 µM). CM-1 (1 μg/ml) and GAPDH (1 μg/ml) primary antibodies were used. Data shown represent three independent experiments. **F** Quantification of p53α expression of MCF-7-LeGO and MCF-7- Δ40p53 cell extracts treated with vehicle (water), CHX (40 µg/mL), MG132 (10 µM) and/or DOX (1 µM). Data shown represent three independent experiments. Results are shown as the mean ± SD. Statistical analyses were carried out using two-way ANOVA followed by Sidak’s post-test. Results were considered significant at *p* < 0.05; *p < 0.05, **p < 0.01, ***p < 0.001, ****p < 0.0001.

The above results show that Δ40p53 knockdown significantly increased apoptosis and pro-apoptotic gene expression; thus, we hypothesised that endogenously expressed Δ40p53 may alter p53α expression and activation following DNA damage. To investigate this, p53α protein expression was examined. There was a time-dependent increase in p53α expression in all MCF-7 sublines when treated with either drug, except for cells overexpressing Δ40p53 (MCF-7-Δ40p53 cells; Fig. 4A). In MCF-7-Δ40p53 cells, elevated p53α was observed at the basal level and its expression at the protein level did not change following DNA-damage (Fig. 4A).

Induction of p53α protein expression following DNA damage in shΔ40p53 cells was significantly enhanced compared to shNT cells (Fig. 4A), which may explain increased apoptosis in these cells. p53α was moderately induced by CIS and DOX in shp53α cells, however it was significantly reduced when compared to shNT cells (Fig. 4A). To confirm p53α protein expression, immunofluorescence was performed for the full-length protein and its phosphorylated form (phosphorylation site: Ser^15^ residue) (Fig. 4B–D). In both cell lines, knockdown of Δ40p53 upregulated the nuclear expression of p53α and its phosphorylated form (Fig. 4B–D), whereas the overexpression of Δ40p53 stabilised p53α levels but did not alter phosphorylation.

The apparent higher stability of p53α in the MCF-7-Δ40p53 subline was further analysed and cells were treated with the translation inhibitor, CHX and/or the proteasome inhibitor, MG132 (Fig. 4E, F). As expected, the levels of p53α decreased in a time-dependent manner following translation inhibition with CHX in LeGO and Δ40p53 cells, however, the extent of reduction in Δ40p53 cells was much less than that observed in the LeGO subline, indicating an increased half-life of p53α when Δ40p53 is overexpressed (Fig. 4E, F).

The effect of translation inhibition on p53α levels was partially attenuated by the addition of MG132 in LeGO cells. In contrast, in Δ40p53 cells, p53α expression remained unaffected by MG132 treatment, supporting previous studies that Δ40p53 overexpression increases p53α’s protein half-life due to modulation of p53α proteasomal degradation [43, 44] (Fig. 4E, F). It should be noted that even though the levels of p53α were increased in the Δ40p53 subline, the expression of Δ40p53 was still comparatively higher (7-fold) than that of p53α (Fig. 4E). As expected, in Δ40p53 cells, Δ40p53 expression was not affected by either treatment (Fig. 4E), supporting previous studies that demonstrated that this isoform exhibits an increased half-life when compared to the full-length protein [5, 30, 37, 43].

### p53α and Δ40p53 co-occur and co-localise after doxorubicin treatment

**Fig. 5:**
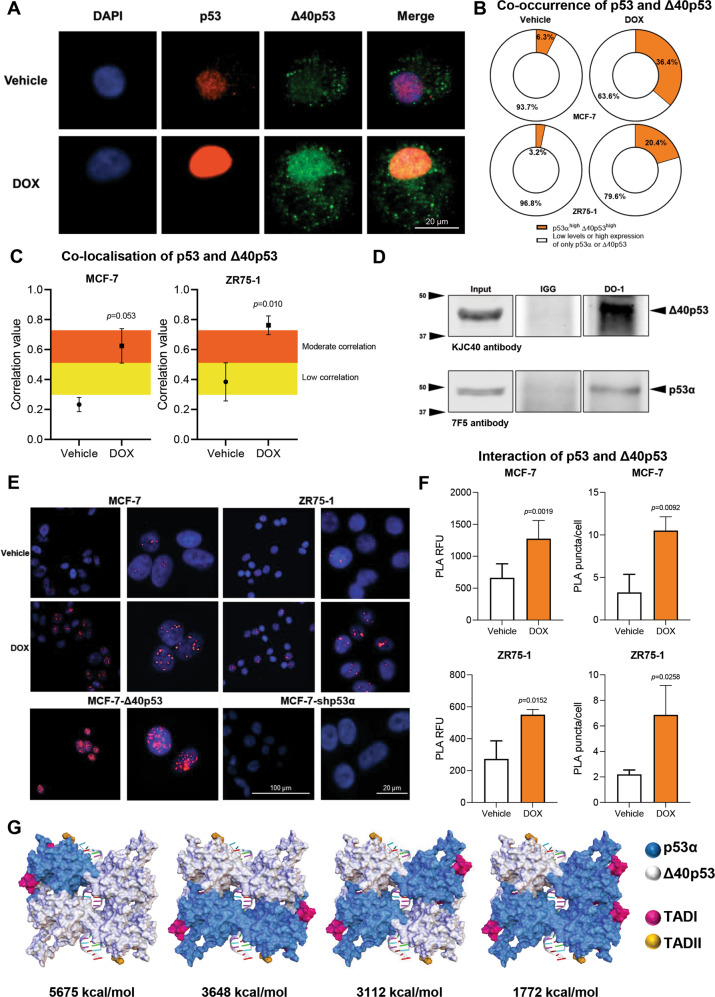
p53 and Δ40p53 co-occur and co-localise after treatment with DOX. **A** Immunofluorescence images of p53α and Δ40p53 staining after treatment with vehicle (water) or DOX (24 h) in the ZR75-1 cell line. DO-1 (1:100) and KJC40 (1:70) primary antibodies were used and cell nuclei were stained with DAPI. **B** Co-occurrence of p53 and Δ40p53 (the presence of both isoforms in the same cell) after treatment with vehicle or DOX in the MCF-7 and ZR75-1 parental cell lines. DO-1 (1:100) and KJC40 (1:70) primary antibodies were used. The mean fluorescence of each stain was used as a threshold and cells with higher or equal fluorescence were considered positive. Data shown represent three independent experiments of three technical replicates. **C** Co-localisation of p53 and Δ40p53 after treatment with vehicle or DOX in the MCF-7 and ZR75-1 parental cell lines. DO-1 (1:100) and KJC40 (1:70) primary antibodies were used. Data shown represent three independent experiments. Spearman’s rank correlation was used for the co-localisation analyses. **D** Co-immunoprecipitation of p53 and Δ40p53 from 500 μg protein extract (from DOX-treated MCF-7-Δ40p53 cells) using 1 μg of anti-p53α (DO-1). Upper panel: the blot was probed for Δ40p53 (KCJ40 antibody; 2.5 µg/mL); lower panel: the blot was probed for p53 (7F5 antibody; 1 µg/mL). For whole membrane, see Supplementary Fig. 3. **E** Representative images of proximity ligation assay (PLA) detection of p53 and Δ40p53 interaction in MCF-7 and ZR75-1 treated with vehicle or DOX. PLA is visualised as red puncta and cell nuclei were stained with DAPI. **F** PLA quantification in MCF-7 and ZR75-1 cells treated with vehicle or DOX. Results are shown as relative fluorescence units (RFU) (left) and the number of PLA puncta per cell count (right). Data shown represent three independent experiments. Results are shown as the mean ± SD. Statistical analyses were carried out using an unpaired *t*-test. Results were considered significant at *p* < 0.05. **G** In silico minimisation potential energy of p53α and Δ40p53 hetero-complexes with DNA. p53α is shown in blue and Δ40p53 in grey. Transactivation domain I (TADI) is shown in magenta (residues 1–40) and transactivation domain II (TADII) is shown in orange (residues 41-61). Each tetramer/DNA complex was energy minimised (CHARMm) with the resultant potential energy (kcal/mol) normalised by the complex with the lowest energy (p53α homo-complex). For sequence alignment and minimisation potential energy calculations of all tetramers see Supplementary Fig. 4.

Δ40p53 retains the oligomerisation domain (OD) and when ectopically expressed, can form hetero-complexes with p53α [5, 26, 28, 33, 45] that can modify p53α-target gene expression. To determine if the endogenously expressed proteins are capable of interacting, the co-occurrence (presence of the two proteins in the same cell), co-localisation (correlation analysis), and proximity ligation assays (PLA) of Δ40p53/p53α complexes before and after DOX treatment was analysed in MCF-7 and ZR75-1 cells. A distinct endogenous expression pattern was observed for each of the isoforms: while p53α was predominantly nuclear, Δ40p53 was expressed as punctate aggregates within the cell, mainly in the cytoplasm (Fig. 5A).

Co-occurrence: At the basal level (in the absence of DOX), 6.3% of MCF-7 cells or 3.2% of ZR75-1 cells highly expressed both p53α and Δ40p53, whereas the majority of cells did not express either protein or p53α and Δ40p53 were expressed alone in individual cells (Fig. 5B). Nevertheless, the number of cells highly expressing both proteins increased around 5-fold following DOX treatment (Fig. 5B).

Co-localisation: The co-localisation of p53α and Δ40p53 was not detected in vehicle-treated cells, where p53α was predominantly localised in the nucleus and Δ40p53 in the cytoplasm. In contrast, following DOX treatment the expression of Δ40p53 became more nuclear (Fig. 5A) and there was a dramatic increase in the co-localisation of p53α and Δ40p53 in both cell lines (Fig. 5A, C).

The physical interaction between these isoforms following DOX treatment was confirmed by co-immunoprecipitation (Fig. 5D, upper panel, lane 3; Supplementary Fig. 3) and PLA (Fig. 5E, F). In vehicle-treated MCF-7 and ZR75-1 cells, an interaction between Δ40p53 and p53α was detected, however, PLA intensity and the number of PLA puncta per cell significantly increased following treatment (*p* < 0.05) (Fig. 5F). These results confirm the endogenous formation of Δ40p53/p53α complexes and the enhancement of this complex formation following DOX treatment.

In order to compare the stability of different compositions of Δ40p53/p53α tetramers, in silico molecular modelling was used, calculating the minimisation potential energies of homo- and hetero-complexes bound to the DNA (Fig. 5G). The p53α homo-complex presented the lowest calculated potential energy (−91674.7 kcal/mol; not shown, this value was set as zero with the relative potential energy of subsequent tetramer configurations reported), suggesting a more favourable assembly and/or higher stability. Although the Δ40p53 homo-complex exhibits structural similarities to p53α at the immediate DNA binding interface, the loss of TAD1 in the Δ40p53 isoform alters the interacting surfaces between the protein subunits, which may impact tetramer formation. The calculated potential energy for the assembled Δ40p53 homo-complex was considerably higher (+7287.8 kcal/mol) than the p53α tetramer (0 kcal/mol), suggesting the formation of a Δ40p53 complex may be less favourable (Supplementary Fig. 4). The calculated potential energies for the hetero-complexes yielded values between the two homo-complexes with higher energies computed for each complex containing an additional Δ40p53 subunit (Fig. 5G). This indicates that the presence of the Δ40p53 isoform may diminish total stability, when incorporated in the p53 complex (Fig. 5G; Supplementary Fig. 4).

### Knockdown of Δ40p53 increases DNA damage at early time points following DOX treatment

**Fig. 6:**
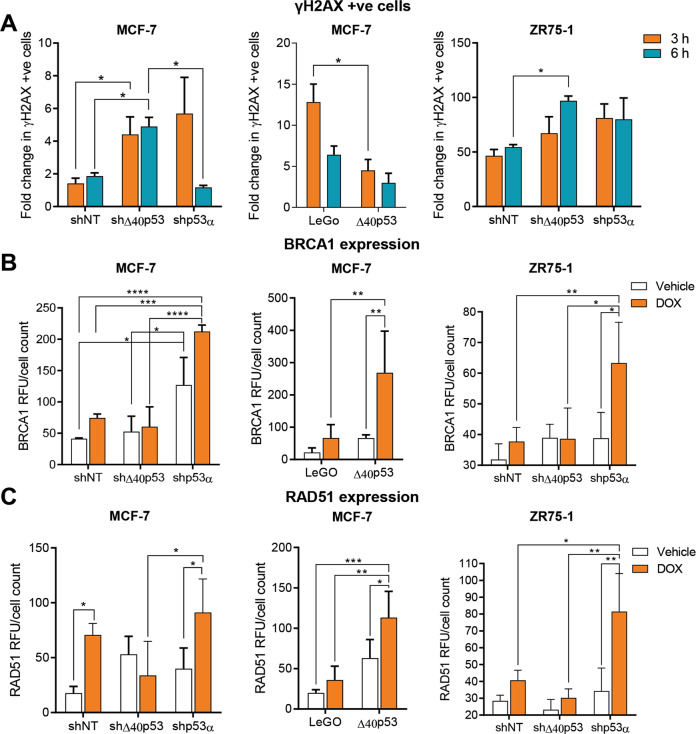
Knockdown of Δ40p53 increases DNA damage following DOX treatment at early time points. **A** γH2AX + ve cells after treatment with DOX (3 and 6 h) and normalised by the 0 h in the MCF-7 (-shNT, -shΔ40p53, -shp53α, LeGO and Δ40p53) and ZR75-1 (-shNT, -shΔ40p53, -shp53α) sublines. γH2AX (1:50) primary antibody was used. Data shown represent three independent experiments of three technical replicates. **B** BRCA1 and **C** RAD51 expression after treatment with vehicle (water) or DOX (3 h) in the MCF-7 (-shNT, -shΔ40p53, -shp53α, LeGO and Δ40p53) and ZR75-1 (-shNT, -shΔ40p53, -shp53α) sublines measured by immunofluorescence. BRCA1 (1:100) and RAD51 (1:100) primary antibodies were used. Data shown represent three independent experiments of three technical replicates. Results are shown as the mean ± SD. Statistical analysis were carried out using two-way ANOVA followed by Sidak’s post-test. Results were considered significant at *p* < 0.05; *p < 0.05, **p < 0.01, ***p < 0.001, ****p < 0.0001.

Given our previous results showing decreased apoptosis induction following DOX in cells expressing high levels of Δ40p53, we next determined if this isoform could impair the DOX-induced DDR. The mechanisms of DOX-mediated cell death are generation of reactive oxygen species, formation of DNA adducts and entrapment of topoisomerase II (TOPOII), which increases torsional strain and causes DSB [46]. Thus, DSBs were evaluated by the formation of γH2AX foci at the break sites after DOX treatment at different time points. Both shΔ40p53 sublines demonstrated increased DNA damage as judged by γH2AX staining when compared to the shNT sublines after 3 and 6 h of treatment (Fig. 6A). In contrast, the overexpression of Δ40p53 was associated with significantly decreased DNA damage after 3 h (*p* = 0.0180) (Fig. 6A), indicating that Δ40p53 may impair the DDR after DOX exposure. The results were confirmed by comet assays in MCF-7 sublines, where shΔ40p53 exhibited the highest tail moment value compared to shNT and shp53α sublines after 6 h of DOX treatment (Supplementary Fig. 5).

p53 can modulate DNA repair pathways via direct interactions with proteins such as RAD51 and BRCA1 [11, 15]. Since we observed increased DSB formation/signalling when Δ40p53 was knocked down and decreased DNA damage when this isoform was overexpressed, we investigated DSB repair by analysing BRCA1 and RAD51 expression. Increased BRCA1 and RAD51 expression were observed in ZR75-1 and MCF-7 shp53α sublines following DOX treatment (Fig. 6B, C), confirming the regulatory effect of p53α on these proteins. Significantly increased expression of BRCA1 (*p* = 0.0077) and RAD51 (*p* = 0.0018) was also observed in MCF-7-Δ40p53 cells after treatment when compared to the LeGO subline (Fig. 6B, C). In contrast, knockdown of Δ40p53 had no significant effect on the expression of RAD51 or BRCA1 (Fig. 6B, C). After 3 h of DOX treatment, p53α was shown to co-localise with BRCA1 (Supplementary Fig. 6A, B) and RAD51 (Supplementary Fig. 6C, D) in MCF-7 and ZR75-1 parental cells, whereas Δ40p53 did not co-localise significantly with either protein. These results were supported by PLA, where it was possible to detect cells where either Δ40p53 or p53α interacted with BRCA1 or RAD51, however, following DOX, only p53α’s interaction with BRCA1 and RAD51 increased (Supplementary Fig. 6E, F).

### Molecular characterisation of the role of Δ40p53 in the cellular response to doxorubicin

To further characterise the mechanisms driving altered DOX responses in cells expressing modified levels of Δ40p53, sublines were treated with DOX for 24 h and their transcriptome was sequenced. Transcript expression was compared between treated sublines expressing high (MCF-7-Δ40p53) and endogenous (MCF-7-LeGO) levels of Δ40p53; and between treated sublines expressing low levels (shΔ40p53 and shp53α) and endogenous (shNT) levels of either Δ40p53 or p53α. Additional comparisons were made between the molecular profiles of treated and untreated cells of each subline to determine whether differential gene expression was already present at the basal level or the result of enhanced/inhibited expression of a particular gene in response to DOX. Using these comparisons, drivers of differential expression were classified in instances where statistical significance was reached (for detailed information regarding the comparisons that were performed and the genes that were differentially expressed, please refer to Supplementary Tables S2–S6 and Supplementary text). In this analysis, 7,390 genes passed quality control and were included.

#### MCF-7-Δ40p53

**Fig. 7:**
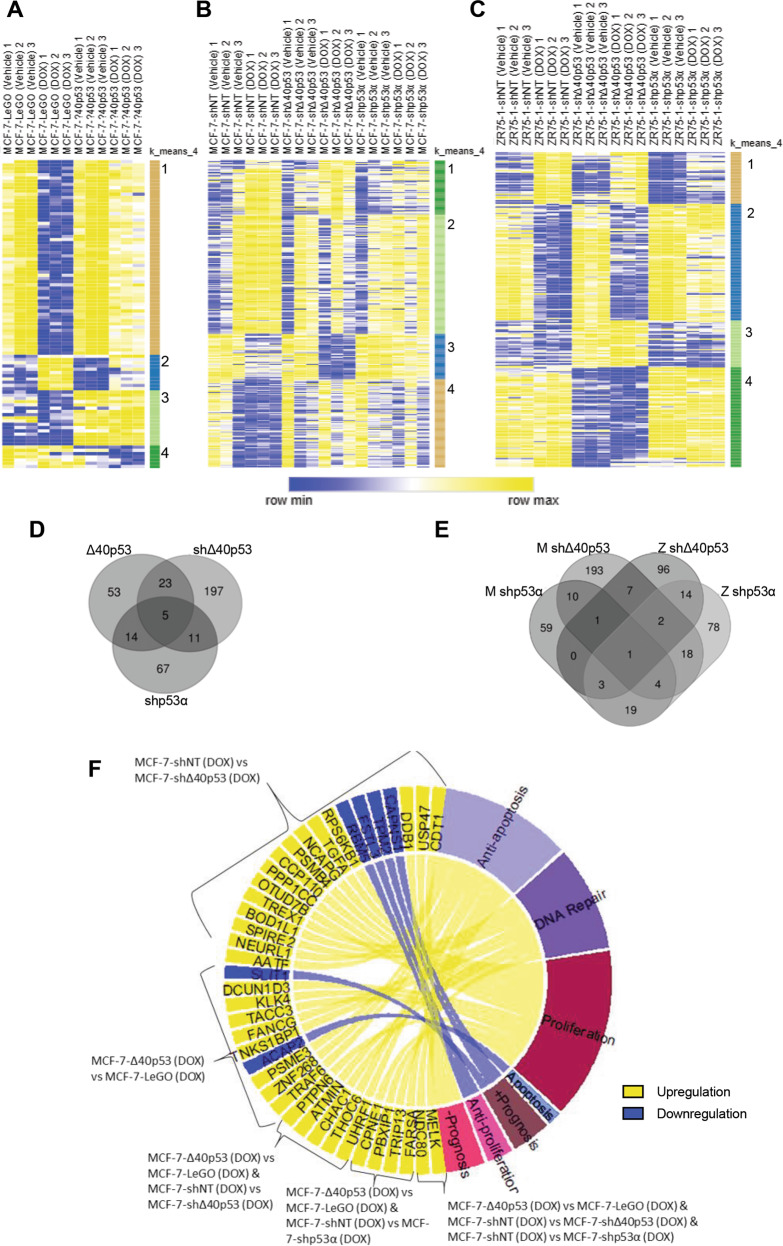
Molecular characterisation of the DOX response in MCF-7 and ZR75-1 sublines. Heatmaps of differentially expressed genes (log_2_ transformed normalised gene counts) between DOX-treated (24 h) **A** MCF-7-Δ40p53 and MCF-7-LeGO cells; **B** MCF-7-shΔ40p53, MCF-7-shp53α, and MCF-7-shNT cells; and **C** ZR75-1-shΔ40p53, ZR75-1-shp53α, and ZR75-1-shNT cells as determined by RNA-seq analysis. Gene clusters are numbered and indicate genes that follow similar patterns. For comprehensive lists of the differentially expressed genes, see Supplementary Table S2–S6. Venn diagrams show common and specific differentially expressed genes (DOX-treated knockdown and overexpression sublines vs. respective controls) between **D** MCF-7 sublines and **E** Δ40p53 and p53α knockdown sublines (MCF-7 and ZR75-1). For a list of genes represented in each Venn diagram field see Supplementary Table S7–S8). **F** Functional annotations of differentially expressed genes that are linked to DOX response in the MCF-7 sublines. Yellow fields indicate genes that exhibit increased expression and blue fields indicate genes that exhibit decreased expression in DOX-treated sublines with increased levels of Δ40p53 or decreased levels of p53α (i.e. MCF-7-Δ40p53 vs MCF-7-LeGO, MCF-7-shNT vs MCF-7-shΔ40p53, and MCF-7-shNT vs MCF-7-shp53α).

RNA-seq revealed 95 differentially expressed genes (DEGs) between DOX-treated Δ40p53 and LeGO cells (1.3%, 95/7390). Differential expression of these genes was driven by the inhibition of DOX-mediated downregulation in Δ40p53 cells (Fig. 7A, cluster 1), the inhibition of DOX-mediated upregulation in Δ40p53 cells (Fig. 7A, cluster 2), or increased expression at baseline that was maintained throughout DOX treatment in Δ40p53 cells (Fig. 7A, cluster 3).

#### MCF-7-shΔ40p53 and MCF-7-shp53α

Two hundred and thirty-six genes (3.2%, 236/7390) were differentially expressed between DOX-treated shΔ40p53 and shNT cells (Fig. 7B, Supplementary Table S3). Knockdown of Δ40p53 inhibited the downregulation of 22 genes (Fig. 7B, cluster 4) and the upregulation of 57 genes (Fig. 7B, cluster 2). p53α knockdown only resulted in 97 DEGs (1.3%, 97/7390), compared to DOX-treated shNT cells (Fig. 7B, Supplementary Table S4). p53α knockdown inhibited the upregulation of 41 genes (Fig. 7B, cluster 1) and the downregulation of 14 genes (Fig. 7B, cluster 3) compared to shNT cells and this inhibition was not evident in shΔ40p53 cells.

#### ZR75-1-shΔ40p53 and ZR75-1-shp53α

In ZR75-1 sublines, 124 genes (1.7%, 124/7930) were differentially expressed between DOX-treated shΔ40p53 and shNT cells (Fig. 7C, Supplementary Table S5). Forty-one downregulated genes already exhibited decreased expression in shΔ40p53 cells at baseline and this was maintained following DOX treatment (Fig. 7C, cluster 4), whereas 19 genes were already upregulated at baseline (Fig. 7C, cluster 3). p53α knockdown resulted in 139 DEGs (1.7%, 139/7930), compared to DOX-treated shNT cells (Fig. 7C, Supplementary Table S6). Compared to Δ40p53 knockdown, p53α knockdown did not affect genes in cluster 3 (Fig. 7C) but inhibited the downregulation of 36 genes (Fig. 7C, cluster 2), and downregulated 11 genes already at baseline (Fig. 7C, cluster 1).

#### Overlap between the MCF-7 and ZR-75-1 sublines and functional annotations

Twenty-eight DEGs in MCF-7-Δ40p53 cells, showed the opposite regulation in MCF-7-shΔ40p53 cells (Fig. 7D). Additionally, there was little overlap between MCF-7 and ZR75-1 knockdown sublines (Fig. 7E), suggesting that different mechanisms govern the altered responses to DOX in each of these sublines (Supplementary Table S7, 8). Functional annotations of the DEGs that are linked to DOX sensitivity in the MCF-7 sublines showed that increased levels of Δ40p53 or decreased levels of p53α (i.e. MCF-7-Δ40p53 vs MCF-7-LeGO, MCF-7-shNT vs MCF-7-shΔ40p53, and MCF-7-shNT vs MCF-7-shp53α) upregulate genes linked to DNA repair, inhibition of apoptosis, proliferation, and worse prognosis (yellow fields); and downregulate genes related to apoptosis, inhibition of proliferation, and better prognosis following DOX treatment (blue fields) (Fig. 7F).

**Fig. 8:**
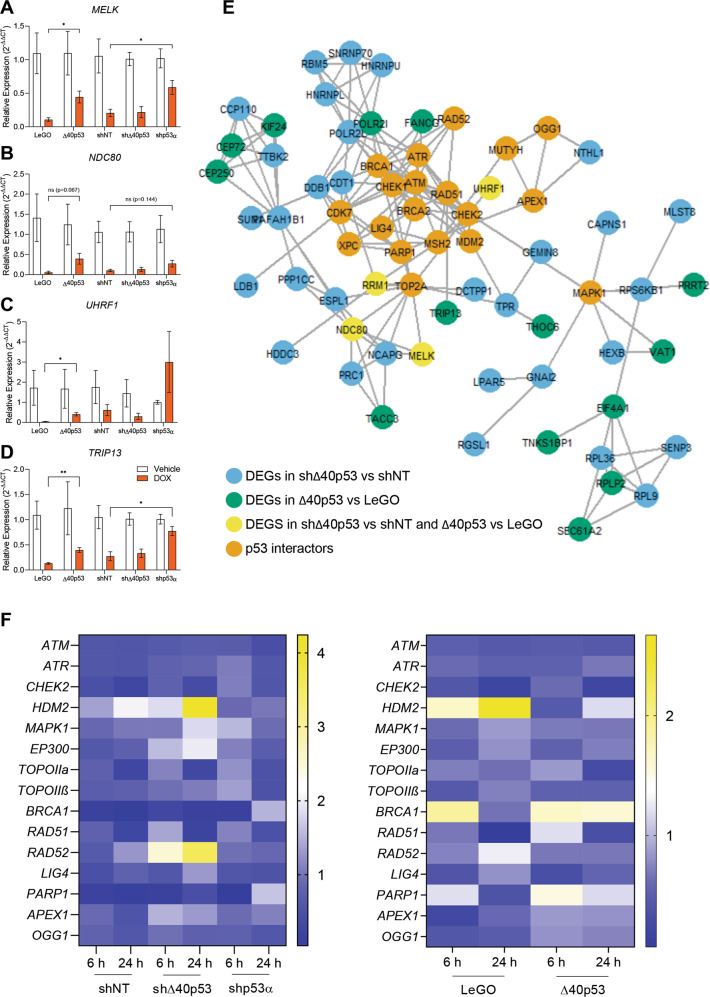
Gene expression of p53 interactors in the MCF-7 sublines. **A**–**D** RNA-seq validation. Gene expression after DOX treatment (24 h) in the MCF-7 (-shNT, -shΔ40p53, -shp53α, LeGO and Δ40p53) sublines. **A** *MELK*, **B** *NDC80*, **C** *UHRF1* and **D** *TRIP13*. Data shown represent three independent experiments of three technical replicates. Results are shown as the mean ± SD and were normalised by the relative expression of the vehicle-treated cells. Statistical analyses were carried out using two-way ANOVA followed by Sidak’s post-test. Results were considered significant at *p* < 0.05; ^*^p < 0.05, ^**^p < 0.01. **E** Interaction between differentially expressed genes (DEGs) and p53 pathway proteins based on STRING data of high confidence (overall interaction score >0.9; see Supplementary Table S9 for breakdown of score). Orange – p53 pathway proteins; Blue: DEGs in DOX-treated MCF-7-shΔ40p53 vs MCF-7-shNT; Green – DEGs in DOX-treated MCF-7-Δ40p53 vs MCF-7-LeGO; Yellow – DEGs in DOX-treated MCF-7-shΔ40p53 vs MCF-7-shNT and MCF-7-Δ40p53 vs MCF-7-LeGO. **F** Heatmaps of mRNA levels of p53 interactors: *ATM*, *ATR*, *CHECK2*, *HDM2*, *MAPK1*, *EP300*, *TOPOIIα*, *TOPOIIβ*, *BRCA1*, *RAD51*, *RAD52*, *LIG4*, *PARP1*, *APEX1* and *OGG1* after 6 or 24 h of DOX treatment and normalised to vehicle-treated cells in the MCF-7 (-shNT, -shΔ40p53, -shp53α, LeGO and Δ40p53) sublines (see Supplementary Fig. 7, 8 for comparison between sublines and statistical analyses). Data shown represent three independent experiments of three technical replicates.

The investigation of each of the individual genes’ function highlighted some important themes. The upregulation of genes linked to the inhibition of apoptosis (e.g. *MELK, NDC80*, *UHRF1*; confirmed by RT-qPCR, Fig. 8A–C), DNA repair (e.g. *TRIP13*, confirmed by RT-qPCR, Fig. 8D), proliferation, and worse prognosis/chemoresistance (*PDK3* [47], *DDB1*, [48], *USP47* [49], *CDT1* [50]) in DOX-treated MCF-7-Δ40p53 cells compared to MCF-7-LeGO cells and in MCF-7-shNT cells compared to MCF-7-shΔ40p53 cells; were common events linked to the alteration of Δ40p53 expression. In confirmation, genes involved in apoptosis, inhibition of proliferation, and better prognosis (*SLIT1* [51], *PTPN4* [52], *TPM2* [53], *CAPNS1* [54]) were downregulated (Fig. 7F), supporting data from functional assays (Fig. 3).

To further evaluate the impact of DEGs in MCF-7 sublines on p53 pathway activity, which is typically activated during the DDR [55], interactions between differentially expressed genes and key p53 pathway proteins were assessed through STRING [56]. Several DEGs detected by RNA-seq were found to interact with key p53 pathway proteins (Fig. 8E, Supplementary Table S9). The expression of these upstream and downstream p53 interactions associated with DOX-related DDR (*ATM, ATR, CHEK2, HDM2, MAPK1, EP300, TOPOIIα, TOPOIIβ, BRCA1, RAD51, RAD52, LIG4, PARP1, APEX1, OGG1*) was analysed by RT-qPCR in MCF-7 sublines after 6 and 24 h of DOX treatment and compared to untreated cells (Fig. 8F). p53α knockdown induced the upregulation of *TOPOIIα, BRCA1*, and *PARP1* following 24 h of treatment when compared to shNT and shΔ40p53 cells (Fig. 8F, Supplementary Fig. 7G, I, M), which may indicate higher activation of DNA repair after treatment. In contrast, no statistically significant differences were found in MCF-7-shΔ40p53 cells (Fig. 8F, Supplementary Fig. 7) when compared to shNT cells.

However, when Δ40p53 was overexpressed a significant increase in *RAD51* expression (Fig. 8F, Supplementary Fig. 8J) was observed, corroborating the protein expression results (Fig. 6B, C) and indicating a specific upregulation of the HR DSB repair pathway in these cells.

## Discussion

The tumour suppressor p53α plays a critical role in maintaining DNA integrity and guiding cellular responses to stress stimuli [57]. We have uncovered novel mechanisms of p53 signalling disruption by Δ40p53 in breast cancer cells following DNA damaging treatment. In this study, overexpression and molecular inhibition were used to modulate the Δ40p53:p53α ratio and investigate the influence of altered Δ40p53 and p53α levels on the DNA-damage response to CIS or DOX in breast cancer cells. Our findings show that Δ40p53 expression is upregulated by the DNA damaging agent DOX, that it promotes G2-arrest and inhibits apoptosis, changing the canonical p53α-driven response to this agent. Moreover, a high Δ40p53:p53α ratio impairs DOX sensitivity by altering protein interactions following DOX, repressing DOX-mediated changes in gene expression, and ultimately driving cells towards DNA repair and survival.

While a low Δ40p53:p53α ratio supported p53’s canonical function, leading to an increased proportion of cells in G1 (Fig. 2), accompanied by pro-apoptotic gene expression (Fig. 3E–L), and apoptosis (Fig. 3A, Supplementary Fig. 2H), a high Δ40p53:p53α ratio was found to be associated with a reduction of the G1 population (Fig. 2), diminished apoptotic gene expression (Fig. 3M–O), and enhanced cell survival (Fig. 3B, D) in response to DOX and CIS. A high Δ40p53:p53α ratio was found to increase the G2 population in response to DOX (Fig. 2H), consistent with Δ40p53 inducing G2 arrest in response to endoplasmic reticulum stress [33]. Differences in cell cycle regulation may be mediated through *GADD45* (mediates G2), which has been found to be upregulated by Δ40p53 yet downregulated by p53α [31].

Altered regulation of apoptotic gene expression and subsequent cell death is likely to be related to the lack of a TAD1 in Δ40p53. It has been previously described that TAD1-mutated p53 (p53^L25Q,W26S^), similar to Δ40p53, is unable to regulate the transcription of *CDKN1A*, *NOXA*, and *PUMA*, and cannot promote cell cycle arrest or apoptosis after acute DNA damage [58, 59], indicating that full-length p53 is required to drive p53’s canonical responses (in this case apoptosis) to DNA-damaging agents [60].

Beyond the regulation of specific pro-apoptotic genes, a high Δ40p53:p53α was found to block the transcriptional program typically initiated by DOX treatment (Fig. 7A, cluster 1 and 2). While Δ40p53’s ability to impair transcriptional activation of p53’s target genes at baseline and following treatment with DNA-damaging agents has been previously reported in overexpression models [5, 29]; the finding that Δ40p53 can also impair transcriptional repression is novel, and suggests a more comprehensive deregulation of cell fate following DNA damage. Functional annotations of the RNA-seq results showed that a high Δ40p53:p53α ratio was associated with DNA repair, inhibition of apoptosis, proliferation, and worse prognosis (Fig. 7F), supporting altered cell fate in response to DOX in cells with elevated Δ40p53:p53α ratios.

Inhibition of transcriptional activation or repression in the presence of elevated Δ40p53 may be driven by (I) the interaction of Δ40p53 and p53α, with both isoforms shown to co-localise and interact following DNA damage treatment in parental cells (Fig. 5C–F). In this instance, the presence of Δ40p53 in the oligomers may decrease their stability when bound to DNA (Fig. 5G; Supplementary Fig. 4); therefore, elevated levels of Δ40p53, and its subsequent incorporation into hetero-tetramers could account for impaired p53 transcriptional function (Fig. 3) and interaction with cofactors. Additionally, by a different mechanism, (II) high levels of Δ40p53 may contribute to misfolded-p53 aggregates [41] since Δ40p53 modulates p53α expression through decreased proteasomal degradation [43, 44] and downregulates *HDM2* [43] (Fig. 4, Fig. 8F, Supplementary Fig. 8D), further altering p53’s activities. Alternatively, (III) PTMs of Δ40p53 and/or electrostatic and hydrophobic interactions between the hetero-tetramer and further downstream signalling, are likely to be significantly impaired, since many proteins interact with TAD1 in a context-dependent fashion following DNA damage [60–64], and hence, as shown by our study, p53-dependent transcription of target genes is compromised when Δ40p53 is highly expressed (Fig. 3M–P). Finally, (IV) with Δ40p53 able to bind to some p53 REs [65, 66], Δ40p53 homo-tetramers may occupy p53 REs and thus, prevent p53α binding and transactivation/repression. All these hypotheses may account for the repression of the canonical p53α-mediated transcriptional program (activation and repression) following DOX, when Δ40p53 is expressed at levels that exceed p53α, leading to a non-canonical response to DOX-induced genomic stress. It is highly likely that Δ40p53 exerts these alterations via a combination of the mechanisms described above. Our current model suggests that the primary function of Δ40p53 in relation to p53 is as a transcription repressor of p53-transcriptional activity in regulating a set of genes involved in the response to DNA damage. In support of this, genes found highly expressed following both Δ40p53 overexpression and p53α knockdown such as *MELK*, *NDC80*, *UHRF1*, *TRIP13*, and *RAD51*, have been found to be dysregulated when p53 is deleted or truncated [67–71], suggesting a loss-of-function-like phenotype. However, further analysis is needed to identify how Δ40p53 represses transcriptional regulation.

The reduced DNA damage when Δ40p53 was highly expressed may be the result of increased DNA repair, supported by increased expression of BRCA1 and RAD51 following DOX (Fig. 6B, C, Fig. 8F, Supplementary Fig. 8I, J). Interestingly, BRCA1 or RAD51 did not co-localise with Δ40p53 and no differences were found in PLA following DOX (Supplementary Fig. 6), suggesting that unlike p53α this isoform may not efficiently form complexes with these proteins and thus exert less control over DNA repair pathways. It was shown that p53α‘s involvement and regulatory activities in the HR pathway can be mediated via interactions with RAD51, RAD54 [17], and BRCA1 [72], independently of its transactivation activity [73, 74]. Even though Δ40p53 maintains the interaction residues with these proteins, it lacks some activation residues of p53 such as the phosphorylation of Ser^15^ by upstream kinases, which is required for HR suppression mediated by p53 [75]. We cannot exclude that there might be other factors associated with the lack of co-localisation or PLA results between Δ40p53 and DNA repair proteins, such as upregulation of other downstream proteins or the formation of misfolded-p53 aggregates as previously mentioned. The analyses shown here are by no means exhaustive and to fully define Δ40p53 protein interactions further analysis is needed. Nevertheless, these results suggest that in addition to inhibiting the transcriptional repression of p53-target genes (in this case *RAD51*), Δ40p53 may also alter the physical interaction of p53 complexes with signalling proteins.

The deregulated DNA repair in MCF-7-Δ40p53 cells may cause hyper-recombination, which could lead to genomic instability and the accumulation of DNA mutations. Our data suggest that the Δ40p53 subline has accumulated more damaged chromosome fragments compared to the LeGO subline, as demonstrated by an increase in the number of micronuclei in this subline (Supplementary Fig. 9). This result supports the hypothesis of deregulated HR when Δ40p53 is overexpressed. Interestingly, amid the DSB repair genes analysed (*RAD51, RAD52*, and *LIG4*), only *RAD51* was upregulated, showing that a high Δ40p53:p53α ratio specifically upregulates the HR pathway, contrasting to the Δ113p53/Δ133p53 isoform, which promotes all three DNA DSB repair pathways [76].

Overall, our results indicate that Δ40p53 is upregulated following DOX, forms complexes with p53α, stabilises p53, and impairs p53’s activation and transactivation of target genes. A high Δ40p53:p53α ratio alters the DDR in breast cancer cells, and to some extent, Δ40p53 behaves similar to TAD1-truncated p53, where p53 loss-of-function is evident in the lack of cell cycle, DNA repair and apoptosis regulation. Our findings are consistent with the repression of p53-transcriptional activities and impaired protein interactions, leading to prevention of apoptosis after DOX treatment and inhibition of DOX-mediated changes in gene expression. Moreover, inhibiting the expression of Δ40p53 resulted in enhanced apoptosis, suggesting a potential therapeutic benefit of a co-therapy that combines Δ40p53 silencing with DNA-damaging chemotherapies used in breast cancer treatment. The influence of Δ40p53 levels on other p53-dependent pathways remains to be defined, but most likely Δ40p53 alters p53 function in a context and cell signal-specific fashion.

## Methods

### Cell lines

The oestrogen receptor-positive human breast cancer cell lines MCF-7 and ZR75-1, expressing wild-type p53 (WTp53), were kindly provided by Professor Christine Clarke (Westmead Millennium Institute, The University of Sydney, Australia) and Dr Judith Weidenhofer (The University of Newcastle, Australia), respectively. The cell lines were authenticated by the Australian Genome Research Facility as previously described [42]. MCF-7 cells stably overexpressing Δ40p53α via the lentiviral LeGO vector, as well as the empty-vector controls have been previously described [42]. Knockdown sublines (-shNT, -shΔ40p53, and -shp53α) were established by transduction of MCF-7 or ZR75-1 cells with lentiviral vectors containing short hairpin RNAs (shRNA) against Δ40p53, p53, or a non-targeting control (NT) [42] (Sigma-Aldrich, Castle Hill, NSW, Australia). Each of the sublines was maintained in DMEM (Dulbecco modified Eagle’s medium) supplemented with 10% foetal bovine serum (FBS), insulin (10 μg/ml), L-glutamine (2 mM) (Life Technologies, Mulgrave, VIC, Australia), and puromycin (1 μg/ml) (Sigma-Aldrich) in humidified 5% CO_2_ at 37 ˚C. Cells were routinely tested for mycoplasma according to the manufacturer’s recommendations (MycoAlert PLUS, Lonza, Basel, Switzerland).

### Cell treatments

Cells were seeded into either 96-well plates at 15,000 cells/well (immunofluorescence and proximity ligation assay) and 5000 cells/well (apoptosis and senescence assays), ultra-low attachment 96-well plates at 4000 cells/well (spheroid assay), 24-well plates at 1 × 10^5^ cells/well (viability assay), or 6-well plates at 5 × 10^5^ cells/well (mRNA, protein analysis, comet assay, and cell cycle analysis). Cells were treated the following day with vehicle (water) or physiologically relevant concentrations of CIS (10 μM) or DOX (1 μM) and/or cycloheximide (CHX; 40 µg/mL) and/or MG132 (10 µM) (Sigma-Aldrich).

### Immunofluorescence

Cells were fixed with 3.7% formaldehyde (Sigma-Aldrich) in phosphate-buffered saline (PBS) for 10 min, then permeabilised in 0.1% Triton-X-100 in PBS for 5 min at room temperature and non-specific antibody binding sites were blocked using 3% FBS in PBS for 30 min. After blocking, cells were incubated for 1 h with primary antibodies: mouse-anti-human-γH2AX, 1:50 dilution (Merck, Macquarie Park, NSW, Australia; #05-636); mouse-anti-human-BRCA1, 1:100 dilution (Life Technologies; #MA1-23164); mouse-anti-human-RAD51, 1:100 dilution (Life Technologies; #MA1-23271); mouse-anti-P-p53 (S15), 1:400 dilution (Cell Signaling Technology, Danvers, MA, USA; #9286); rabbit-anti-human-p53 7F5 (which detects the first 50 amino acids of p53), 1:800 dilution (Cell Signaling Technology; #2527); rabbit-anti-human-KJC40 (which detects the Δ40p53 epitope MDDLMLSPDDIEQWFTE with specific PTMs; antibody validation: Supplementary Fig. 1), 1:70 dilution (developed by J.C. Bourdon, The University of Dundee, Scotland), and mouse-anti-human-DO-1 (which detects the sequence ^20^SDLWKL^25^of the TAD1 of p53), 1:100 dilution (Merck; #MABE327). Then, the cells were washed three times with PBS and incubated for 1 h with secondary antibodies: goat-anti-mouse-Alexa-Fluor 594, 1:30 dilution (Life Technologies; #R37121) and/or goat-anti-rabbit-Alexa 488, 1:500 dilution (Life Technologies; #A11034) or goat-anti-rabbit-Alexa 594, 1:500 dilution (Life Technologies; #A11037). All antibodies were diluted in blocking solution at room temperature. Each well was then stained with DAPI (300 nM in PBS) to detect nuclei. Images were obtained using the Cytation3 cell imager multi-mode reader (BioTek, Winooski, VT, USA) using 10x and 40x objectives. Negative controls were included for each primary and secondary antibody and for DOX (Supplementary Fig. 1). Four images were collected per well maintaining exposure and contrast settings. Images were analysed using the Gen5 software (BioTek) and ImageJ for co-localisation. Images identification was blinded to the investigator.

### Proximity ligation assay

To detect protein interactions, proximity ligation assays were performed using Duolink In Situ Red Starter Kit Mouse/Rabbit (Sigma-Aldrich) following the manufacturer’s recommendations. Briefly, cells were fixed with 3.7% formaldehyde (Sigma-Aldrich) in PBS for 10 min, then permeabilised in 0.1% Triton-X-100 in PBS for 5 min at room temperature. After blocking, cells were incubated for 1 h at room temperature with primary antibodies (DO-1 and KJC40; 7F5 and BRCA1 or RAD51; KJC40 and BRCA1 or RAD51; details including dilutions of each antibody are described above). Then, cells were incubated for 1 h at 37 ˚C with the proximity ligation assay probes, followed by ligation of the probes for 30 min at 37 ˚C and amplification for 100 min at 37 ˚C. After the final washes, the cells were stained with DAPI. Images were obtained using the Cytation3 cell imager multi-mode reader (BioTek) using 20x and 40x objectives. Negative controls, which lacked the primary antibodies but contained the PLA probes or that contained the primary antibodies but lacked the PLA probes or that contained only one primary antibody and the PLA probes were included. Biological controls were also included: MCF-7-Δ40p53 cells were used as a positive control and MCF-7-shp53α were used as a negative control to assess the Δ40p53 and p53α interaction. Four images were collected per well maintaining exposure and contrast settings. Approximately 40 cells were evaluated per triplicate. Images were analysed using Gen5 software (BioTek) and the relative fluorescence units, as well as the number of puncta per cell, were recorded. Images identification was blinded to the investigator.

### Cell cycle analysis

After 24 h treatments, cells were trypsinised and fixed with cold 70% ethanol for 1 h at 4 °C. Fixed cells were washed twice with cold PBS and stained with FxCycleTM PI/RNase Staining Solution for 15 min at room temperature, protected from light. Data were acquired on a FACSCantoTM flow cytometer (BD Biosciences, Macquarie Park, NSW, Australia). Five thousand events were collected for each sample. Data were analysed using Kaluza^TM^ software (Beckman Coulter, Indianapolis, IN, USA). Doublets were removed using forward scatter (FSC) height vs FSC area plots. Cell counts were plotted against PI staining intensity and gates were drawn to delineate G1, S, and G2 cell populations. Cell populations at G1, S, and G2 were normalised to untreated cells within the same phase in order to identify the relative changes.

### Apoptosis and viability assays

For MCF-7 cells, IncuCyte Annexin-V red reagent (Essen Bioscience, Ann Arbor, MI, USA) was added to the cells at the time of DOX or CIS treatment at a dilution of 1:200 in PBS. Labelled cells were imaged every 3 h using the IncuCyte Zoom live-cell imaging system (Essen Bioscience). Images were analysed using the IncuCyte Zoom 2016B software. The proportion of Annexin-V positive cells was normalised to the confluence of each image to account for any variability in cell number. Data are shown as the proportion of Annexin-V positive cells at each time point relative to the proportion at 0 h. For ZR75-1 cells, the Trypan blue exclusion assay and Promega RealTime-Glo™ Annexin V Apoptosis assays were used. For Trypan blue, cells were counted after treatment using the automated cell counter Countess II (Life Technologies). Data are shown as the percentage of viable cells for each time point relative to vehicle-treated cells. For apoptosis, 100 μl of media containing vehicle, CIS or DOX was mixed with diluted Detection Reagent including Annexin V-SmBiT, Annexin V-LgBiT, and CaCl_2_. The Cytation3 cell imager multi-mode reader (BioTek) was used to read the luminescent values every 24 h starting from 0 h. Data are shown as the proportion of Annexin-V luminescence at each time point relative to 0 h time point.

### Cell spheroids assay

Cells were seeded in ultra-low attachment 96-well plates (Corning, NY, United States), centrifuged for 5 min at 200 x *g* and incubated for two days to allow the formation of cell spheroids. Medium containing vehicle or DOX was then added to each well. After four days of treatment, half of the media was replenished with further treatment-containing media and spheroids were incubated for an additional four days, then, half of the media was replenished with complete drug-free media and spheroids were incubated for an additional four days. The spheroids were imaged every second day using the Cytation3 cell imager (BioTek) for spheroid size measurement, relative to their size prior to treatment. Spheroid viability was assessed using the CellTiter-Glo 3D assay (Promega, United States) on the 14^th^ day according to the manufacturers’ instructions. Data are shown as the percentage of spheroid viability relative to vehicle-treated spheroids.

### Alkaline comet assay

Alkaline comet assays were performed as previously described by Singh et al. [77], with minor modifications. Briefly, 30 μL of cell suspension was mixed with 70 μL low-melting point agarose, spread on an agarose pre-coated microscope slide and placed at 4 °C for 10 min to allow solidification. Cells were lysed in a high concentration salt and detergent solution (2.5 M NaCl, 100 mM Na_2_EDTA, 10 mM Tris with 1% Triton-X 100%, and 10% DMSO) for 24 h. Slides were removed from the lysis solution and washed three times with PBS. Next, cells were exposed to alkali conditions (300 mM NaOH, 1 mM Na_2_EDTA, pH >13, 15 min, 4 °C). Following DNA unwinding, the slides were subjected to electrophoresis at 1.7 V/cm for 15 min using a Sub-Cell DNA Gel Electrophoresis Apparatus (Bio-Rad, Gladesville, NSW, Australia). Slides were neutralised and stained with GelGreen® (Biotium, Fremont, CA, USA) and mounting media (Agilent Technologies, Mulgrave VIC, Australia). Slide images were captured using a Cytation3 with a 20x objective, and all slides were analysed using the freeware TriTek CometScore. The tail moment was measured (Tail moment=tail lengthx% of DNA in the tail) for 100 cells on two slides per biological triplicate. All results were compared to vehicle treatments.

### Gene expression

#### RNA isolation

Total RNA was extracted from all cell lines using TRIzol RNA purification reagent (Life Technologies) following manufacturer’s recommendations. The RNA yield was determined by the Qubit RNA BR (broad range) Assay Kit (Life Technologies) on a Qubit 2.0 Fluorometer (Life Technologies), following manufacturer recommendations. RNA integrity was assessed using the Agilent 4200 Tapestation System and the Agilent High Sensitivity (HS) RNA ScreenTape assay (Agilent Technologies). An RNA integrity number (RIN) was given for each sample. Samples with a RIN ≥ 7 were used for RNA-seq described below.

Reverse transcription quantitative polymerase chain reactions (RT-qPCR)

500 ng of total RNA was reverse transcribed into complementary DNA (cDNA) using the High-Capacity Reverse Transcription kit with RNase inhibitor (Life Technologies), as per the manufacturer’s instructions. No template RNA and no reverse transcriptase controls were included. PCR efficiency curves were used to select an appropriate cDNA dilution within the linear detection range (around 10 ng/reaction). TaqMan Advanced Master Mix (Life Technologies) and TaqMan Gene Expression assays for p53 target genes (including primers and probes: Supplementary Table S1; Life Technologies), *Δ40p53* (as previously described [38]), *TP53*, and *GAPDH* as an endogenous control were used. Relative expression was calculated using the 2^-ΔΔCt^ method. For *MELK*, *NDC80*, *UHRF1* and *TRIP13*, expression is shown as relative expression. For the other primers, expression is shown as fold-change compared to relative expression of vehicle-treated samples.

#### RNA-sequencing

RNA libraries were generated using the Illumina stranded mRNA library preparation kit. Pooled libraries were loaded onto a 500/550 High Output Flow Cell (single-end, 75 cycles) and run on a NextSeq 500 system (Illumina, San Diego, CA, USA). FASTQ files were produced by BaseSpace (Illumina) and mapped to Human GRCh37 Assembly using STAR [78]. Differential expression was computed in DESeq2. Genes of ≥50 counts, log_2_(fold change) ≥ |1| with a false discovery rate (FDR) adjusted *p*-value ≤ 0.05 were considered differentially expressed.

#### Gene Set enrichment analysis

Gene set enrichment analysis (GSEA) was carried out by Enrichr [79] using GO Biological Process 2018. Biological processes were based on 100 most relevant genes (where more than 100 DEGs where searched) and processes with an adjusted *p* value of <0.05 were deemed significant. Processes with odds ratios >2 or <0.5 were considered as up- or down-regulated processes respectively.

#### Protein interaction network

Interactions between differentially expressed genes and other p53 and DDR genes were assessed at the protein level using the STRING database [56]. Only interactions of high confidence (interaction score >0.9) were considered.

### Protein expression

#### Sample preparation

Cell pellets were lysed in 1% NP-40 lysis buffer (50 mM Tris-HCl, 150 mM NaCl, 1% NP-40, pH 8.0, 1 x Mini complete Protease Inhibitor cocktail tablet per 10 ml) and sonicated using the Bioruptor sonicator (Diagenode, Liège, Belgium). Protein concentrations were assessed using a Bradford assay (Bio-Rad) according to the manufacturer’s instructions and the absorbance was read on an Implen NanoPhotometer (Implen, München, Germany).

#### Co-immunoprecipitation

p53 was immunoprecipitated from 500 μg protein extract (from DOX-treated MCF-7-Δ40p53 cells) using 1 μg of anti-p53 (DO-1) and the Dynabeads Protein G Immunoprecipitation Kit (Life Technologies) according to manufacturer’s recommendations. Mouse IgG Isotype Control (Life Technologies) was used to estimate non-specific binding of primary antibodies.

#### Immunoblot assays

Proteins were separated by sulphate dodecyl sulphate-polyacrylamide gel electrophoresis (SDS-PAGE) as previously described [42]. The membrane was blocked with Casein Blocking Buffer (Millennium Science, Mulgrave VIC, Australia) or Intercept™ PBS Blocking Buffer (LI-COR Biosciences, Lincoln, NE, USA) at room temperature for 1 h. The following primary antibodies were diluted in blocking buffer: CM-1 (pan-p53 polyclonal antibody, which recognises epitopes located between amino acids 1 and 393), 1 μg/ml; KJC40, 2.5 μg/ml (The University of Dundee, Scotland); DO-1, 1 μg/ml (Merck); 7F5, 1 μg/ml (Cell Signaling Technology); mouse GAPDH, 1 μg/ml (Calbiochem, San Diego, CA, USA; #CB1001); rabbit GAPDH, 1 μg/ml (Abcam, Melbourne, VIC, Australia; #ab128915), and added to the membrane overnight (4 °C, rocking). Diluted secondary antibodies (1–5 μg/ml; LI-COR Biosciences; #926-32210 and #926-68023) in blocking buffer were added and allowed to bind on a rocker for at least 1 h at room temperature. Bands were visualised and quantitated using an Odyssey CLx fluorescent imager (LI-COR Biosciences) relative to the loading control (GAPDH).

### In silico analysis

The stability of p53α and Δ40p53 hetero-complexes with DNA was evaluated by molecular docking. Briefly, the structures of the p53 complexes were generated by homology modelling using the target sequence UniProtKB P04637, residues 94–356 and template structures: PDB ID 4MZR (Chains A–D, residues 94-358), PDB ID 3EXJ (Chain A, residues 98–291), PDB ID 4IBU (Chains A, B, residues 94–293), and PDB ID 1OLG (Chain A, residues 319–360). Discovery Studio v.18.1 (Biovia) was used, to create the multiple sequence alignments, homology models, loop modelling of residues 14-60 using PDB ID 2K8F (Chain, residues 14–60), tetrameric complexes, and energy minimisations. Combinations of p53 and Δ40p53 subunits were docked into tetramer complexes with DNA (ZDOCK), using PDB ID 4MZR as the template. Each tetramer/DNA complex was then energy minimised (CHARMm) with the resultant potential energy (kcal/mol) reported.

### Statistical analysis

Unpaired student t-tests were performed for two comparisons and one-way ANOVA or two-way ANOVA for multiple comparisons, corrected for multiple comparisons using the Dunnett’s test, Tukey’s test (one-way), or Sidak’s test (two-way). All results are the mean of three independent experiments (*n* of technical replicates are indicated in the figure legends), and error bars represent the standard deviation (SD). All statistical analyses were performed using GraphPad Prism v. 6.0 (GraphPad Software, La Jolla, CA, USA). An adjusted *p*-value of <0.05 was considered statistically significant.

## Supplementary information

Supplementary material

Supplementary Table S2

Supplementary Table S3

Supplementary Table S4

Supplementary Table S5

Supplementary Table S6

Supplementary Table S9

Reproducibility Checklist

Original Data File

Manuscript describing the production and validation of the MCF-7 and ZR75-1 sublines.

## Data Availability

The datasets generated during and/or analysed during the current study are available from the corresponding author on reasonable request.
