# Supplementary material for: Alterations in the p53 isoform ratio govern breast cancer cell fate in response to DNA damage
Source: Cell Death Dis. 2022 Oct 28;13(10):907. doi: 10.1038/s41419-022-05349-9 (PMC9616954; doi:10.1038/s41419-022-05349-9)
Supplement: Supplementary file 1 — Supplementary material [file 41419_2022_5349_MOESM1_ESM.pdf]

Supplementary Figures

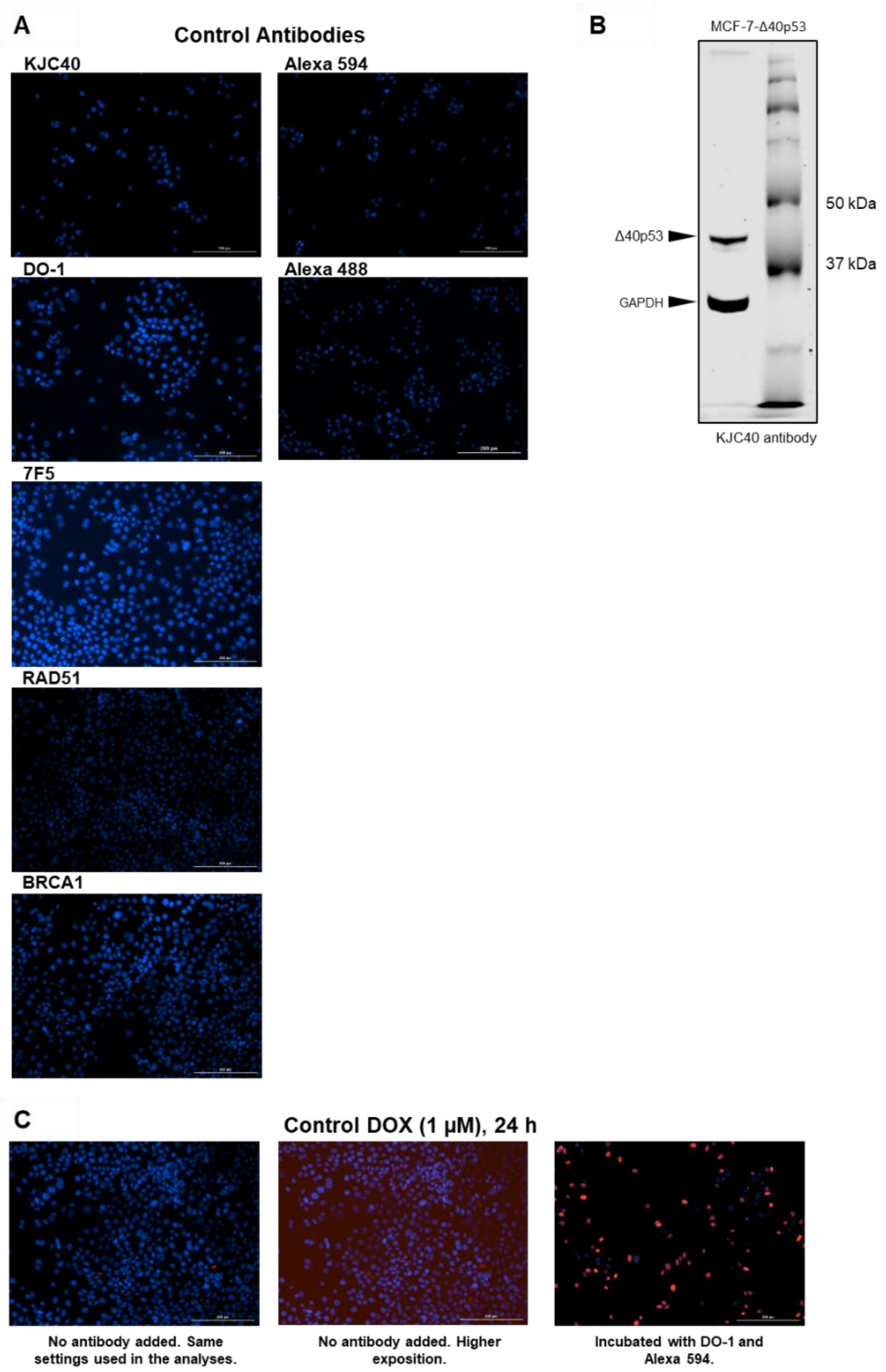

Figure 1: Antibodies validation. (A) Immunofluorescence images when only the primary antibodies (KJC40, DO-1, 7F5, RAD51 or BRCA1) were added or only the secondary antibodies (goat-anti-rabbit-Alexa 594 or goat-anti-mouse-Alexa 555) were added. Cell nuclei were stained with DAPI. **(B)** Representative western blot membrane of MCF-7-Δ40p53 subline extract (30 μg) probed for Δ40p53 and GAPDH. The KJC40 antibody was used at a dilution of 2.5 μg/mL for western blots and GAPDH was used at a dilution of 1 μg/mL. **(C)** Immunofluorescence images when only DOX was added for 24 h using the same settings used for the immunofluorescence analyses or with a higher exposure or when both the DO-1 primary antibody and Alexa 594 secondary antibody were used.

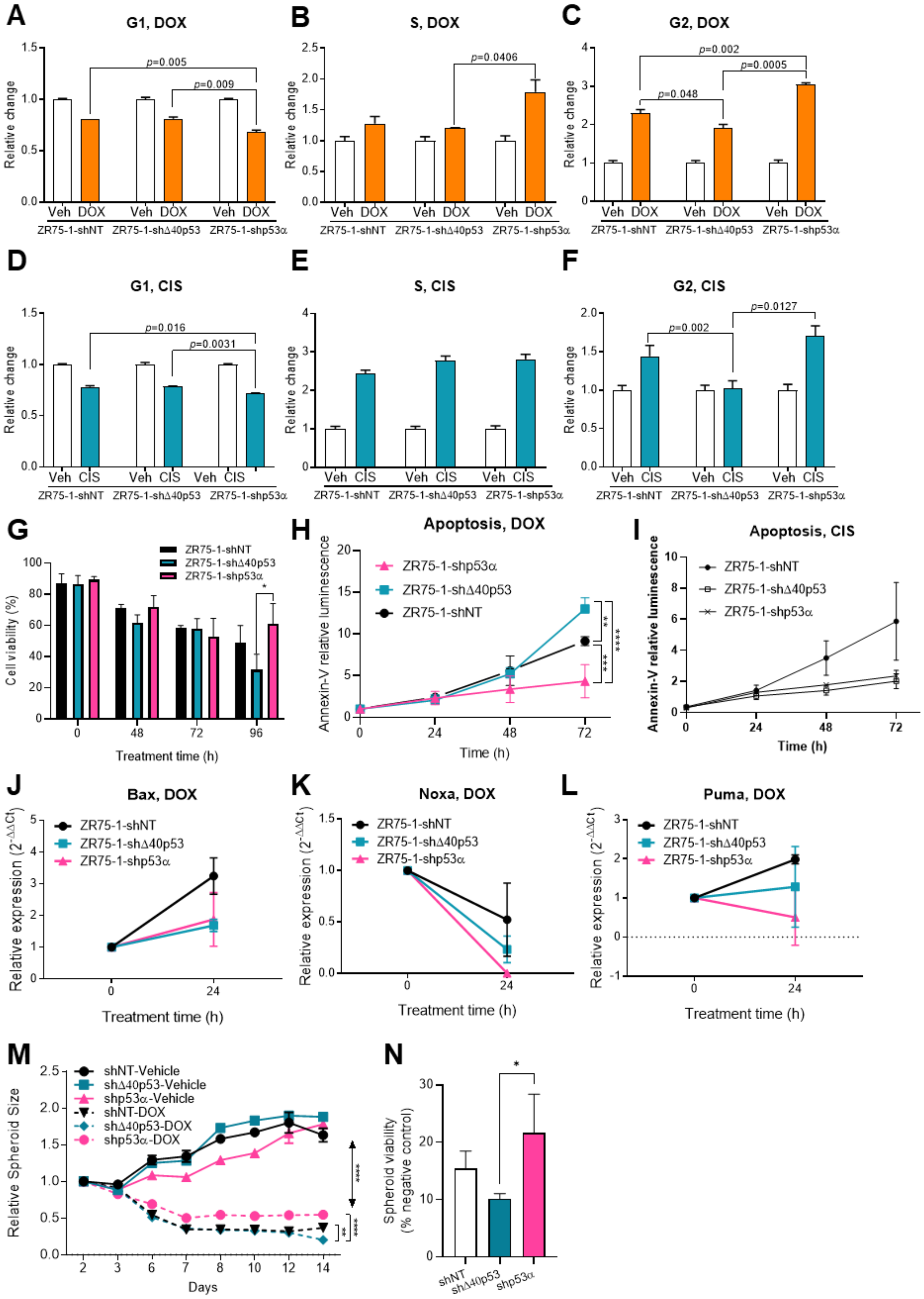

Figure 2: Modulation of Δ40p53 following DOX treatment impairs cell response in ZR75-1 sublines. Relative change in **(A)** G1-phase, **(B)** S-phase and **(C)** G2-phase in the ZR75-1 (-shNT, -shΔ40p53, -shp53α) sublines

treated with DOX. Data shown represent three independent experiments of three technical replicates. Relative change in **(D)** G1-phase, **(E)** S-phase and **(F)** G2-phase in the ZR75-1 (-shNT, -shΔ40p53, -shp53α) sublines treated with CIS. Data shown represent three independent experiments of three technical replicates. **(G)** Cell viability of ZR75-1 (-shNT, -shΔ40p53, -shp53α) sublines analysed by Trypan blue staining following DOX treatment. Data shown represent three independent experiments of three technical replicates. **(H)** Annexin-V relative luminescence normalised to luminescence of 0 h time point in ZR75-1 (-shNT, -shΔ40p53, -shp53α) sublines following DOX or **(I)** CIS treatment. Data shown represent three independent experiments of three technical replicates. mRNA levels of **(J)** *BAX*, **(K)** *NOXA* and **(L)** *PUMA* in response to DOX normalised to vehicle-treated cells in ZR75-1 (-shNT, -shΔ40p53, -shp53α) sublines. Data shown represent three independent experiments of three technical replicates. **(M)** Spheroid size normalised to size prior to treatment with DOX and **(N)** spheroid viability normalised to vehicle-treated spheroids in ZR75-1 (-shNT, -shΔ40p53, -shp53α) sublines. Data shown represent three independent experiments of four technical replicates. Results are shown as the mean ± SD. Statistical analyses were carried out using unpaired t-test **(A-F)**, two-way ANOVA followed by Dunnett's post-test **(G)**, one-way ANOVA followed by Tukey's post-test **(H, I, N),** or two-way ANOVA followed by Sidak's post-test **(J-M)**. Results were considered significant at $p < 0.05$; *$p < 0.05$; **$p < 0.01$, ***$p < 0.001$, ****$p < 0.0001$.

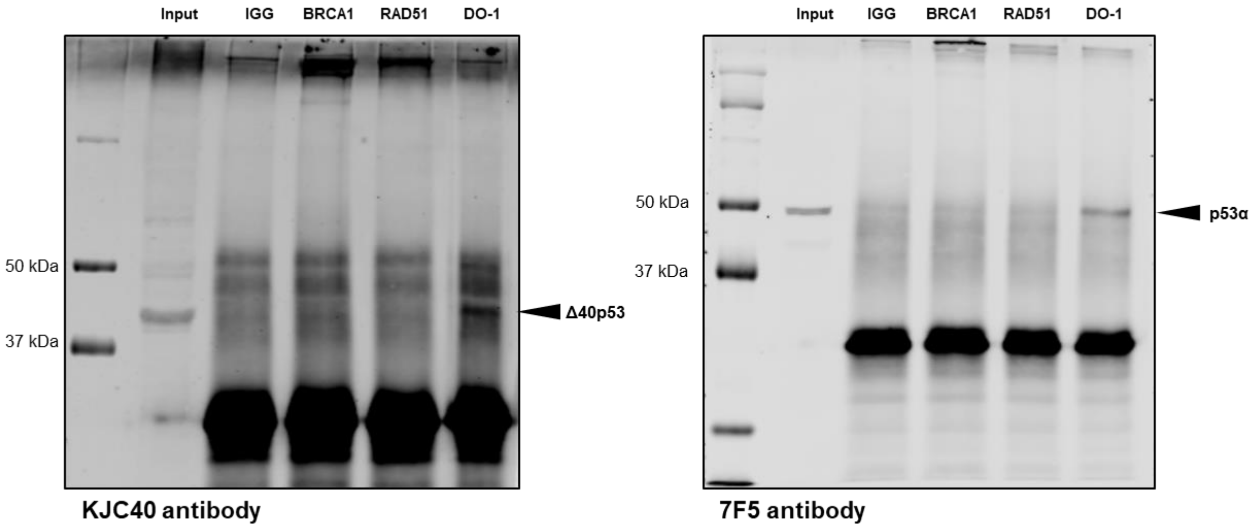

Figure 3: Co-immunoprecipitation of p53 and Δ40p53 after DOX treatment. Left: Whole membrane of the co-immunoprecipitation of p53 from 500 µg protein extract (from MCF-7-Δ40p53 cells) using 1 µg of anti-p53α (DO-1). The blot was probed for Δ40p53 using the KCJ40 antibody at a dilution of 2.5 µg/mL. Right: experiment validation; whole membrane of the co-immunoprecipitation of p53 from 500 µg protein extract (from MCF-7-Δ40p53 cells) using 1 µg of anti-p53 (DO-1). The blot was probed for p53α using the 7F5 antibody at a dilution of 1 µg/mL.

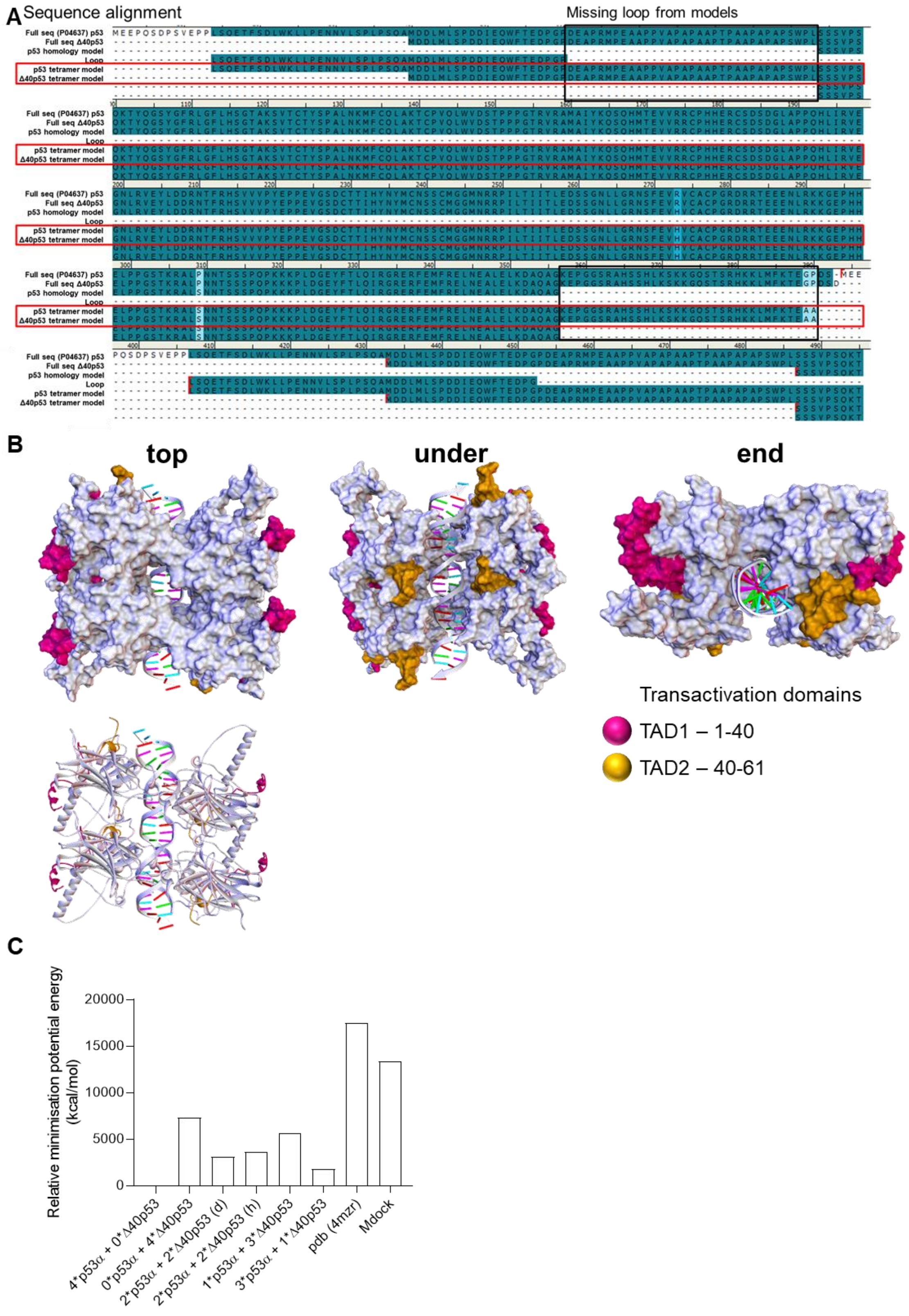

Figure 4: *In silico* structure and assembly of p53α and Δ40p53 heterocomplexes with DNA. (A,B) The structures of the p53 complexes were generated by homology modelling. p53α is shown in blue and Δ40p53 in grey. The transactivation domain I (TADI) is shown in magenta (residues 1–40) and the transactivation domain II (TADII) is shown in orange (residues 41-61). The structures of the p53 complexes were generated by homology modelling using the target sequence UniProtKB P04637, residues 94–356 and template

structures: PDB ID 4MZR (Chains A–D, residues 94-358); PDB ID 3EXJ (Chain A, residues 98–291); PDB ID 4IBU (Chains A, B, residues 94–293) and PDB ID 1OLG (Chain A, residues 319–360). Discovery Studio v18.1 (Biovia) was used to create the multiple sequence alignments, homology models, loop modelling of residues 14-60 using PDB ID 2K8F (Chain, residues 14–60), tetrameric complexes and energy minimisations. Combinations of p53 and Δ40p53 subunits were docked into tetramer complexes with DNA (ZDOCK), using PDB ID 4MZR as the template. **(C)** Each tetramer/DNA complex then was energy minimised (CHARMm) with the resultant potential energy (kcal/mol) normalised by the complex with the lowest energy (p53α homo-complex).

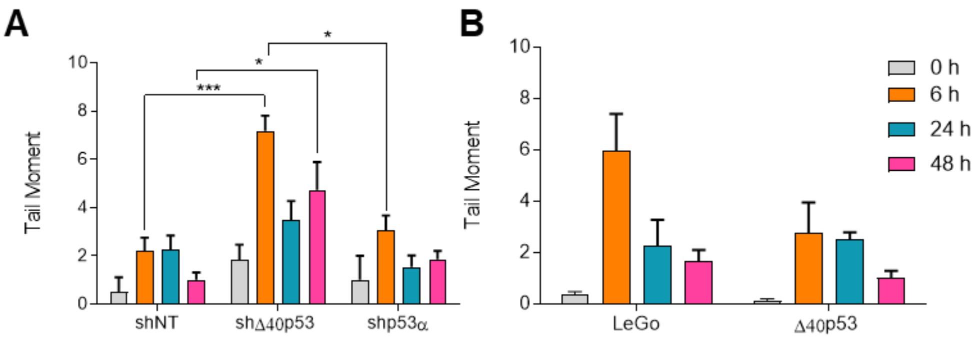

Figure 5: Comet assay of MCF-7 sublines after DOX treatment. Tail moment of **(A)** MCF-7-shNT, MCF-7-shΔ40p53 and MCF-7-shp53α and **(B)** MCF-7-Δ40p53 and MCF-7-LeGO sublines treated with DOX for 6, 24 and 48 h. Data shown represent three independent experiments of two technical replicates. Results are shown as the mean ± SD. Statistical analyses were carried out using two-way ANOVA followed by Sidak's post-test. Results were considered significant at $p < 0.05$; *$p < 0.05$, ***$p < 0.001$.

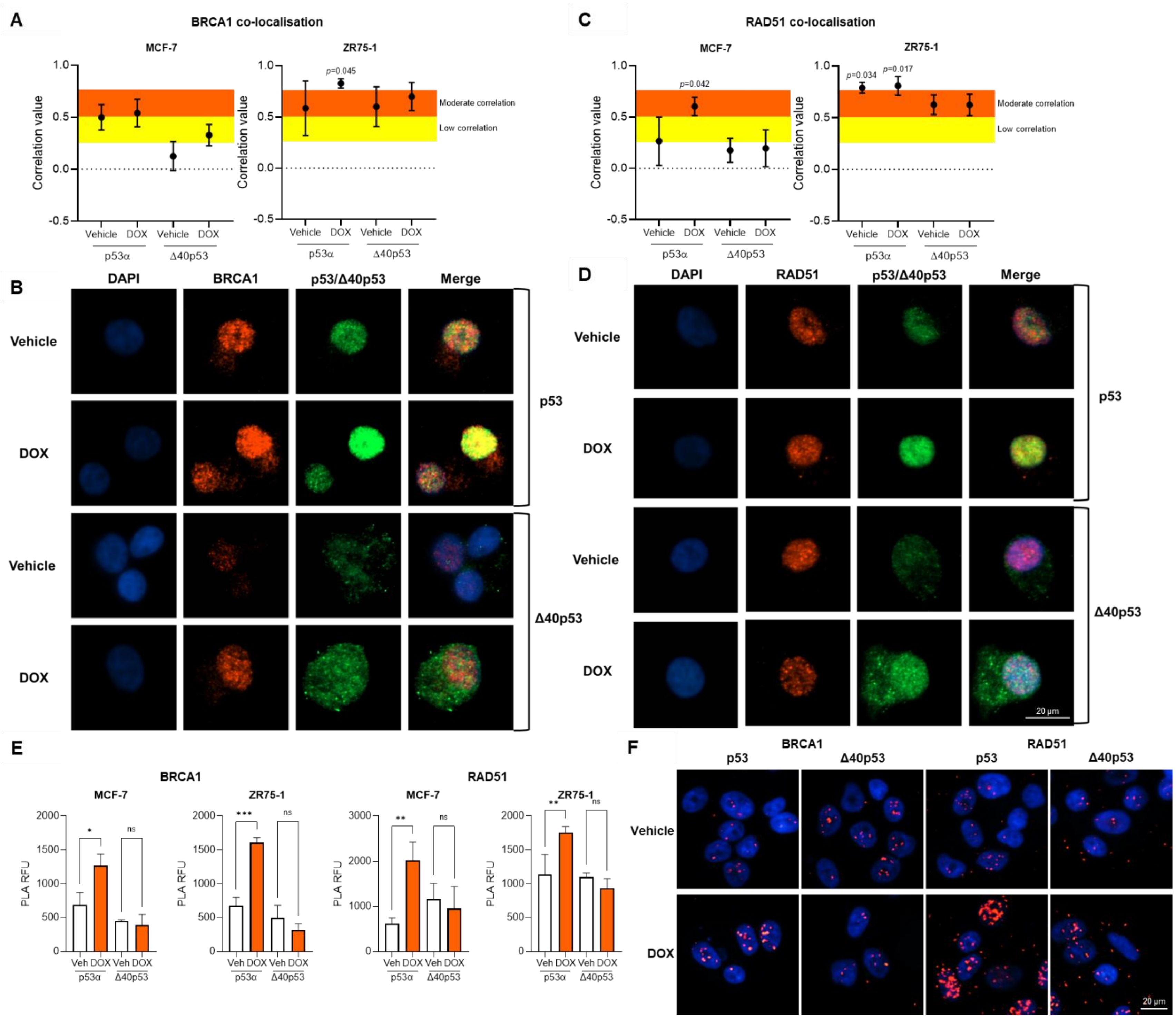
A
BRCA1 co-localisation
MCF-7
ZR75-1
Correlation value
p=0.045
Moderate correlation
Low correlation
Vehicle DOX Vehicle DOX
p53α
Δ40p53
C
RAD51 co-localisation
p=0.042
p=0.034
p=0.017
B
DAPI
BRCA1
p53/Δ40p53
Merge
Vehicle
DOX
p53
Δ40p53
D
RAD51
20 µm
E
BRCA1
RAD51
PLA RFU
Veh DOX Veh DOX
ns
F

Figure 6: Interaction of p53 and Δ40p53 with BRCA1 or RAD51. (A) Co-localisation of p53 or Δ40p53 with BRCA1 after treatment with vehicle (water) or DOX (3 h) in the MCF-7 and ZR75-1 parental cell lines. Data shown represent three independent experiments. **(B)** Immunofluorescence images of p53α or Δ40p53 and BRCA1 staining after treatment with vehicle or DOX (3 h) in the ZR75-1 cell line. BRCA1 (1:100), 7F5 (1:800) and KJC40 (1:70) primary antibodies were used and cell nuclei were stained with DAPI. **(C)** Co-localisation of p53 or Δ40p53 with RAD51 after treatment with vehicle or DOX (3 h) in the MCF-7 and ZR75-1 parental cell lines. Data shown represent three independent experiments. **(D)** Immunofluorescence images of p53α or Δ40p53 and RAD51 staining after treatment with vehicle or DOX (3 h) in the ZR75-1 cell line. RAD51 (1:100), 7F5 (1:800) and KJC40 (1:70) primary antibodies were used and cell nuclei were stained with DAPI. Results are shown as the mean ± SD. Spearman's rank correlation was used for the co-localisation analyses. **(E)** Proximity ligation assays (PLA) quantification in MCF-7 and ZR75-1 cells treated with vehicle or DOX (3 h). Results are shown as relative fluorescence units (RFU). Data shown represent three independent experiments. Results are shown as the mean ± SD. Statistical analyses were carried out using one-way ANOVA followed by Tukey's post-test. Results were considered significant at $p < 0.05$; *$p < 0.05$, **$p < 0.01$, ***$p < 0.001$. **(F)** Representative images of PLA detection of either p53 or Δ40p53 interaction with BRCA1 or RAD51 in MCF-7 treated with vehicle or DOX. PLA is visualised as red puncta and cell nuclei were stained with DAPI.

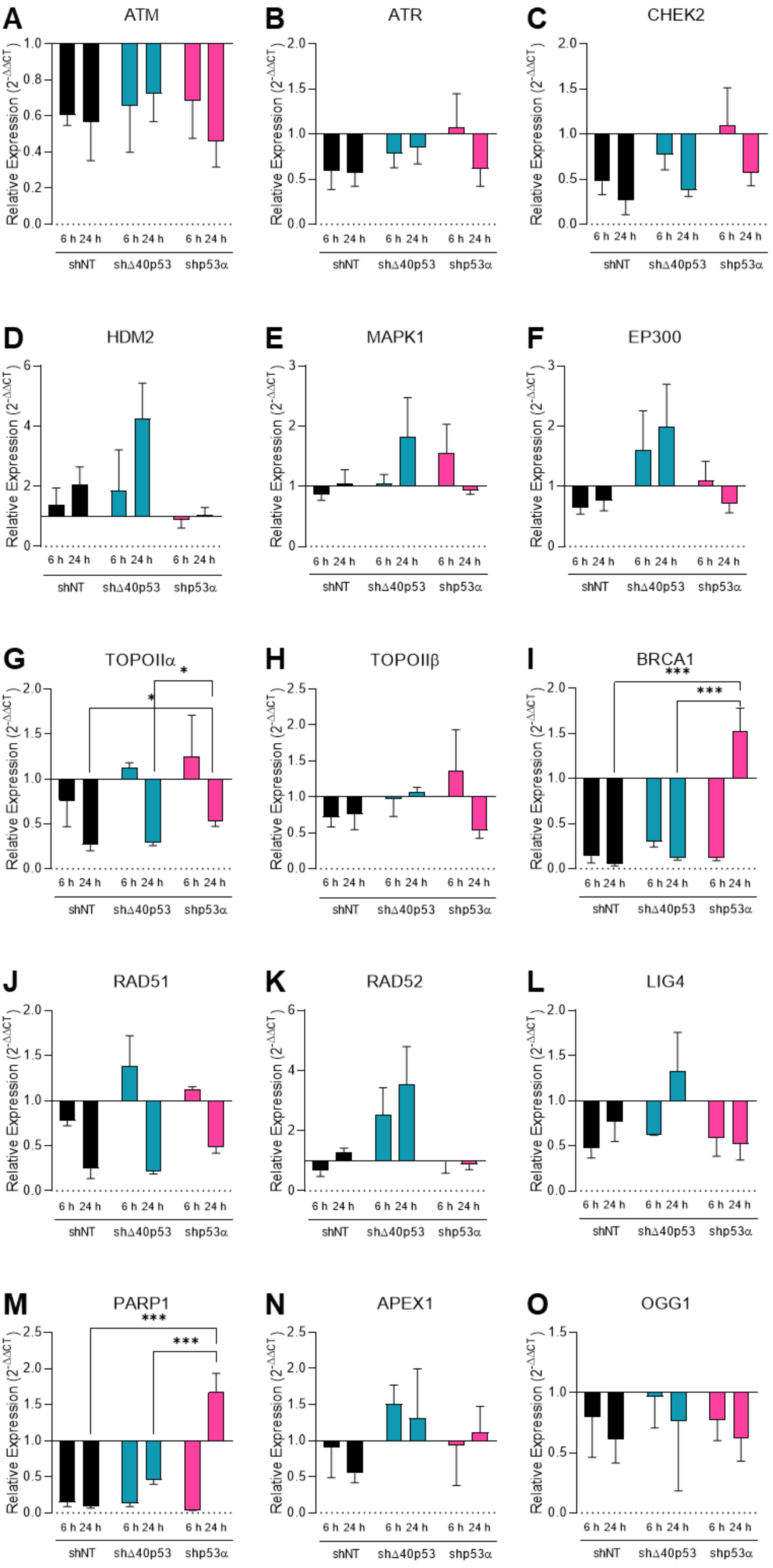

A ATM
B ATR
C CHEK2
D HDM2
E MAPK1
F EP300
G TOPOIIα
H TOPOIIβ
I BRCA1
J RAD51
K RAD52
L LIG4
M PARP1
N APEX1
O OGG1
Relative Expression (2^-ΔΔCT)
6 h 24 h
shNT
shΔ40p53
shp53α
*
***

Figure 7: Gene expression of p53 interactors after DOX treatment in the MCF-7-shNT, MCF-7-shΔ40p53 and, MCF-7-shp53α sublines. mRNA levels of **(A)** *ATM*, **(B)** *ATR*, **(C)** *CHECK2*, **(D)**, *HDM2*, **(E)** *MAPK1*, **(F)** *EP300*, **(G)** *TOPOIIα*, **(H)** *TOPOIIβ*, **(I)** *BRCA1*, **(J)** *RAD51*, **(K)** *RAD52*, **(L)** *LIG4*, **(M)** *PARP1*, **(N)** *APEX1* and **(O)** *OGG1* after 6 or 24 h of DOX treatment as determined by real-time RT-PCR. Data shown represent three independent experiments of three technical replicates. Results are shown as the mean ± SD and were normalised by the relative expression of the vehicle-treated cells. Statistical analyses were carried out using two-way ANOVA followed by Sidak's post-test. Results were considered significant at $p < 0.05$; *$p < 0.05$, ***$p < 0.001$.

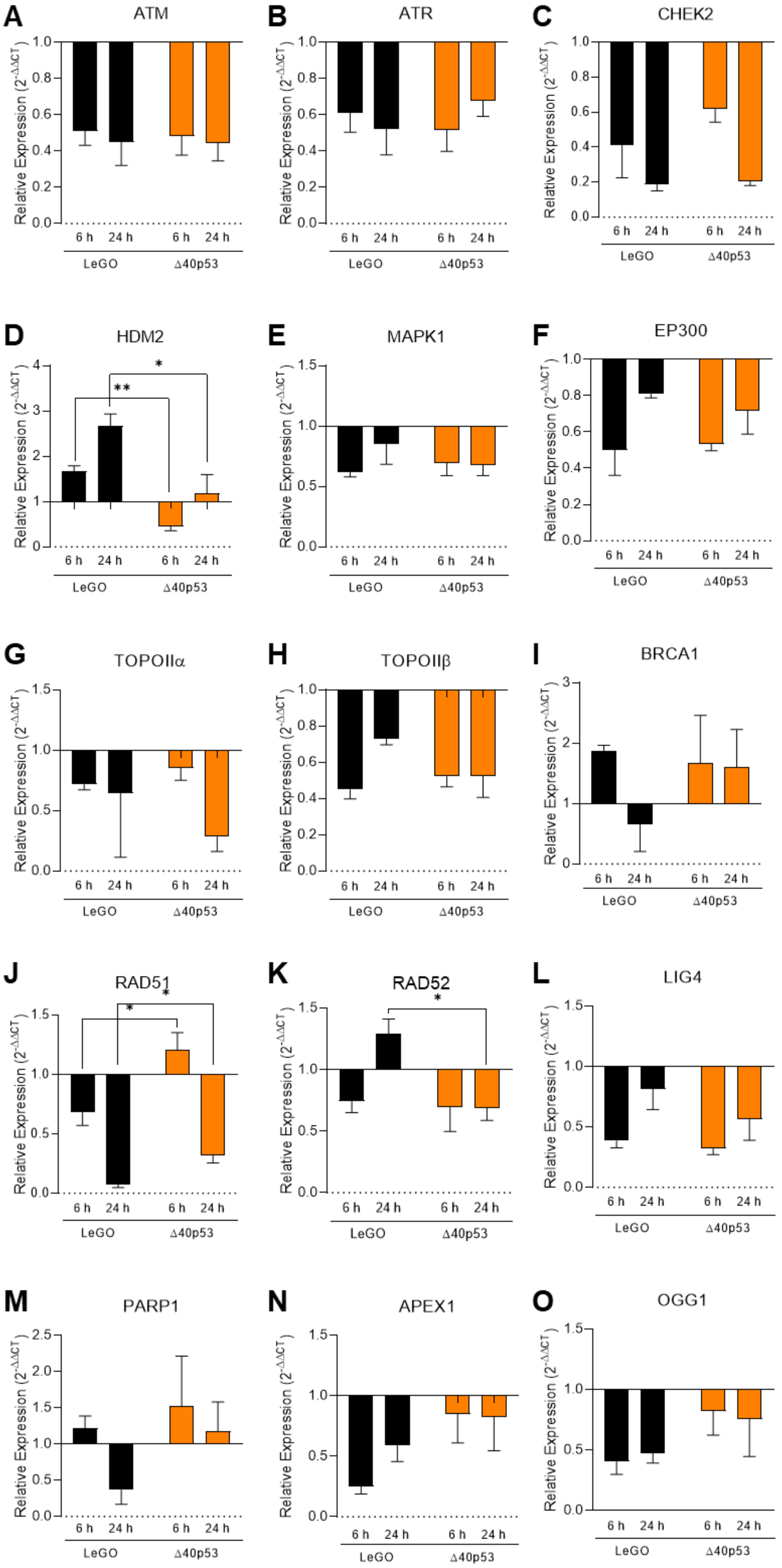
A
ATM
B
ATR
C
CHEK2
D
HDM2
E
MAPK1
F
EP300
G
TOPOIIα
H
TOPOIIβ
I
BRCA1
J
RAD51
K
RAD52
L
LIG4
M
PARP1
N
APEX1
O
OGG1
Relative Expression (2^-ΔΔCT)
6 h
24 h
LeGO
Δ40p53
*
**

Figure 8: Gene expression of p53 interactors after DOX treatment in the MCF-7-LeGO and MCF-7-Δ40p53 sublines. mRNA levels of **(A)** *ATM*, **(B)** *ATR*, **(C)** *CHECK2*, **(D)**, *HDM2*, **(E)** *MAPK1*, **(F)** *EP300*, **(G)** *TOPOIIα*, **(H)** *TOPOIIβ*, **(I)** *BRCA1*, **(J)** *RAD51*, **(K)** *RAD52*, **(L)** *LIG4*, **(M)** *PARP1*, **(N)** *APEX1* and **(O)** *OGG1* after 6 or 24 h of DOX treatment as determined by real-time RT-PCR. Data shown represent three independent experiments of three technical replicates. Results are shown as the mean ± SD and were normalised by the relative expression of the vehicle-treated cells. Statistical analyses were carried out using two-way ANOVA followed by Sidak's post-test. Results were considered significant at $p < 0.05$; *$p < 0.05$, **$p < 0.01$.

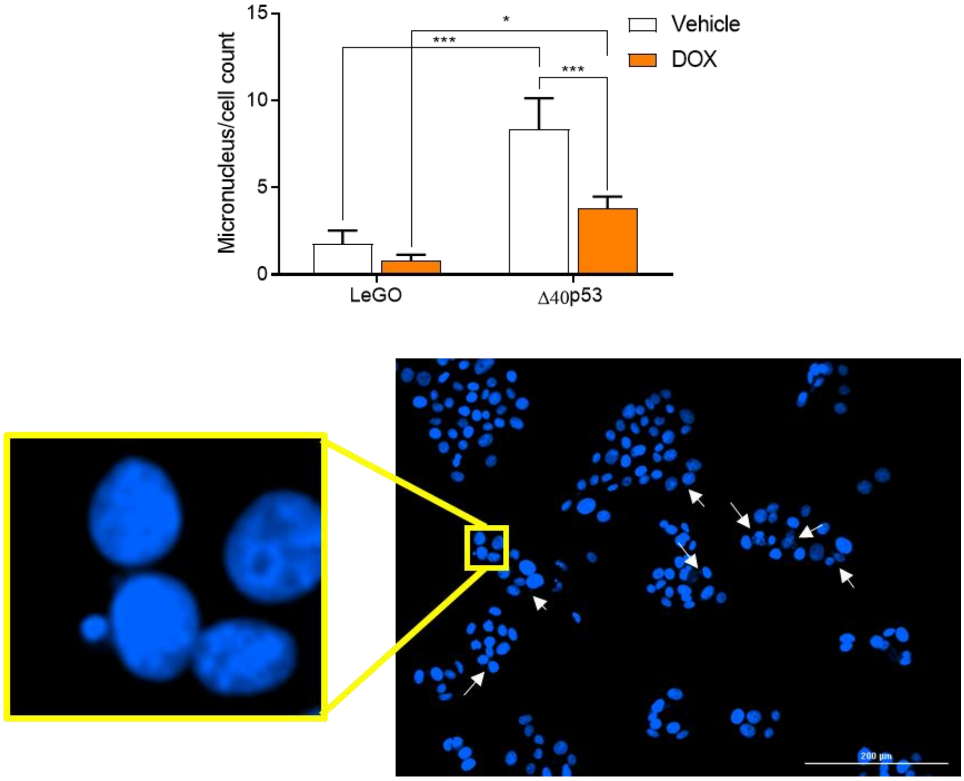

Figure 9: Micronucleus evaluation. MCF-7-LeGO and MCF-7-Δ40p53 sublines were treated with DOX for 24 h and stained with DAPI. Images were obtained using the Cytation3 cell imager multi-mode reader (BioTek, USA) using 10x and the number of micronuclei was analysed using the Gen5 software. Data shown represent three independent experiments. Results are shown as the mean ± SD. Statistical analyses were carried out using two-way ANOVA followed by Sidak's post-test. Results were considered significant at $p < 0.05$; *$p < 0.05$, ***$p < 0.001$. Representative image demonstrates MCF-7-Δ40p53 cells without treatment with DOX (white arrows show micronuclei and yellow box shows the zoomed image).

Supplementary Tables S1, S7, S8

Supplementary Table S1: TaqMan Gene Expression assays (including primers and probes).

Primer	ID
BAX	Hs00180269_m1
NOXA	Hs00560402_m1
PUMA	Hs00248075_m1
TOPOIIβ	Hs00172259_m1
TOPOIIα	Hs06627146_s1
ATR	Hs00992123_m1
ATM	Hs00175892_m1
RAD51	Hs00153418_m1
LIG4	Hs01866071_u1
RAD52	Hs01028879_m1
P300	Hs00914223_m1
OGG1	Hs00213454_m1
CHEK2	Hs00200485_m1
MAPK1	Hs01046830_m1
APEX1	Hs00172396_m1
MDM2	Hs06636310_s1
CDKN1A	Hs00355782_m1
BRCA1	Hs01556193_m1
ATMIN	Hs00796220_s1
MELK	Hs01106438_m1
NDC80	Hs01092161_m1
PTPN6	Hs00169359_m1
TRIP13	Hs01020073_m1
UHRF1	Hs01086727_m1
Δ40p53	As previously described (1)
TP53	Hs01034249_m1
GAPDH	Hs02786624

Supplementary Table S7: Differentially Expressed Genes (DEGs) in Overlapping Venn Diagram Fields for MCF-7 Sublines (Fig. 7D).

**Venn Diagram Fields**	**Number of overlapping DEGs**	**Genes**
Δ40p53 shΔ40p53 shp53α	5	ERN2, ALOXE3, MELK, ZNF681, NDC80
Δ40p53 shΔ40p53	23	CBWD2, AC010618.1, AC092718.3, AP3S2, AC091181.1, BBS12, NDUFA9, HIST1H3G, ATP5MC2, UHRF1, ANHX, IFITM1, AP000911.1, RAB3IL1, SERPINA5, CD99, ANPEP, ATMIN, PTPN6, CHAC1, RRM1, THOC6, BICDL2
Δ40p53 shp53α	14	AC244153.1, AC092718.5, PBXIP1, CPNE1, LRG1, EIF4A1, AC016876.2, FARSA, HNRNPL, ITSN2, SENP3-EIF4A1, TRIP13, VHL, DGCR6
shΔ40p53 shp53α	11	TMEM184A, LINC02318, ZNF16, MAP4K3, RUSC1-AS1, PTPN14, ASIC2, ACOT9, ZSCAN25, PICK1, HEATR6

Δ40p53: DEGs in DOX-treated MCF-7-Δ40p53 vs LeGO.

shΔ40p53: DEGs in DOX-treated MCF-7-shΔ40p53 vs MCF-7-shNT.

shp53α: DEGs in DOX-treated MCF-7-shp53α vs MCF-7-shNT.

Supplementary Table S8: Differentially Expressed Genes (DEGs) in Overlapping Venn Diagram Fields for Knockdown Sublines (Fig. 7E).

Venn Diagram Fields	Number of overlapping DEGs	Genes
M shΔ40p53 M shp53α Z shΔ40p53 Z shp53α	1	ZNF16
M shΔ40p53 M shp53α Z shΔ40p53	1	PICK1
M shΔ40p53 M shp53α Z shp53α	4	ERN2, ALOXE3, MELK, NDC80
M shp53α Z shΔ40p53 Z shp53α	3	LRG1, SERPINB9, HNRNPL
M shΔ40p53 Z shΔ40p53 Z shp53α	2	AC079768.3, GREB1L
M shΔ40p53 M shp53α	10	TMEM184A, LINC02318, ZNF16, MAP4K3, RUSC1-AS1, PTPN14, ASIC2, ACOT9, ZSCAN25, PICK1, HEATR6
M shp53α Z shp53α	19	AC244153.1, AC092718.5, CPNE1, EIF4A1, SYCE2, DOCK8, PRSS50, AL024508.2, F13B, AC016876.2, PRODH, BAALC, PTPN4, FARSA, ITSN2, SENP3-EIF4A1, VHL, CD68, DGCR6
M shΔ40p53 Z shΔ40p53	7	ESPL1, ZNF252P-AS1, LIFR-AS1, NTHL1, ZNF41, PDXK, BCAP29
M shΔ40p53 Z shp53α	18	CBWD2, AC092718.3, DNAJC27-AS1, AC091181.1, BBS12, NDUFA9, SPIRE2, C5orf34, ATP5MC2, UHRF1, SENP3, PGAP3, AP000911.1, CD99, ANPEP, CHAC1, THOC6, BICDL2
Z shΔ40p53 Z shp53α	14	AC118754.1, DCAF11, AC096763.1, DICER1, RBM24, BAIAP3, PPM1K, TNKS1BP1, WWOX, AL022311.1, MYBBP1A, PURPL, TUBB4A, AC037459.2

M shΔ40p53: DEGs in DOX-treated MCF-7-shΔ40p53 vs MCF-7-shNT.

M shΔ40p53: DEGs in DOX-treated MCF-7-shΔ40p53 vs MCF-7-shNT,

Z shΔ40p53: DEGs in DOX-treated ZR75-1-shΔ40p53 vs ZR75-1-shNT.

Z shp53a: DEGs in DOX-treated ZR75-1-shp53α vs ZR75-1-shNT.

Supplementary text

Molecular characterisation of the role of Δ40p53 in the cellular response to doxorubicin

MCF-7-Δ40p53: RNA-seq revealed 95 differentially expressed genes (DEGs) between DOX-treated Δ40p53 and LeGO cells (1.3%, 95/7390). Sixteen DEGs were downregulated in Δ40p53 cells (Fig. 7A, Supplementary Table S2). Differential expression of half of these genes (47/95) was driven by the inhibition of DOX-mediated downregulation in Δ40p53 cells (Fig. 7A, cluster 1). For 8 genes, DOX-mediated upregulation was inhibited in Δ40p53 cells (Fig 7A, cluster 2); and for another 8 genes, differential expression was the result of increased expression at baseline that was maintained throughout DOX treatment in Δ40p53 cells (Fig 7A, cluster 3).

MCF-7-shΔ40p53 and MCF-7-shp53α: Two hundred and thirty-six genes (3.2%, 236/7390) were differentially expressed between DOX-treated shΔ40p53 and shNT cells, including 149 downregulated genes in shΔ40p53 cells (Fig 7B, Supplementary Table S3). Fourteen genes were upregulated in DOX-treated shΔ40p53 compared to untreated cells whilst 43 genes were downregulated in DOX-treated shΔ40p53 compared to untreated cells (Fig. 7B, cluster 1 and 3). Knockdown of Δ40p53 inhibited the downregulation of 22 genes (Fig. 7B, cluster 4) and the upregulation of 57 genes (Fig. 7B, cluster 2). p53α knockdown only resulted in 97 DEGs (1.3%, 97/7390), 53 of which were downregulated, compared to DOX-treated shNT cells (Fig. 7B, Supplementary Table S4). Differences between the knockdown of p53α and Δ40p53 were most prominent at the level of individual genes (Fig. 7D, Supplementary Table S7), yet p53α knockdown also inhibited the upregulation of 41 genes (Fig. 7B, cluster 1) and the downregulation of 14 genes (Fig. 7B, cluster 3) compared to shNT cells and this inhibition was not evident in shΔ40p53 cells.

ZR75-1-shΔ40p53 and ZR75-1-shp53α: In ZR75-1 sublines, 124 genes (1.7%, 124/7930) were differentially expressed between DOX-treated shΔ40p53 cells and shNT cells and more than half of these genes (82/124) were downregulated in shΔ40p53 cells (Fig. 7C, Supplementary Table S5). Half of the downregulated genes (41/82) already exhibited decreased expression in shΔ40p53 cells at baseline and this was maintained following DOX treatment (Fig. 7C, cluster 4). Similarly, 19 genes were already upregulated at baseline (Fig.

7C, cluster 3). One-hundred and thirty-nine genes (1.7%, 139/7930) were differentially expressed between DOX-treated shp53α cells and shNT cells and 47 of these DEGs were downregulated in shp53α cells (Fig. 7C, Supplementary Table S6). Compared to Δ40p53 knockdown, p53α knockdown did not affect genes in cluster 3 (Fig. 7C) but inhibited the downregulation of 36 genes (Fig. 7C, cluster 2). A further 11 genes were already downregulated in shp53α cells at baseline, and this was maintained throughout DOX treatment (Fig. 7C, cluster 1).

References

1. Avery-Kiejda KA, Morten B, Wong-Brown MW, Mathe A, Scott RJ. The relative mRNA expression of p53 isoforms in breast cancer is associated with clinical features and outcome. Carcinogenesis. 2014;35(3):586-96.
