# Supplementary material for: Alterations in the p53 isoform ratio govern breast cancer cell fate in response to DNA damage
Source: Cell Death Dis. 2022 Oct 28;13(10):907. doi: 10.1038/s41419-022-05349-9 (PMC9616954; doi:10.1038/s41419-022-05349-9)

Original western blots

Figure 1B

Top image (MCF-7):

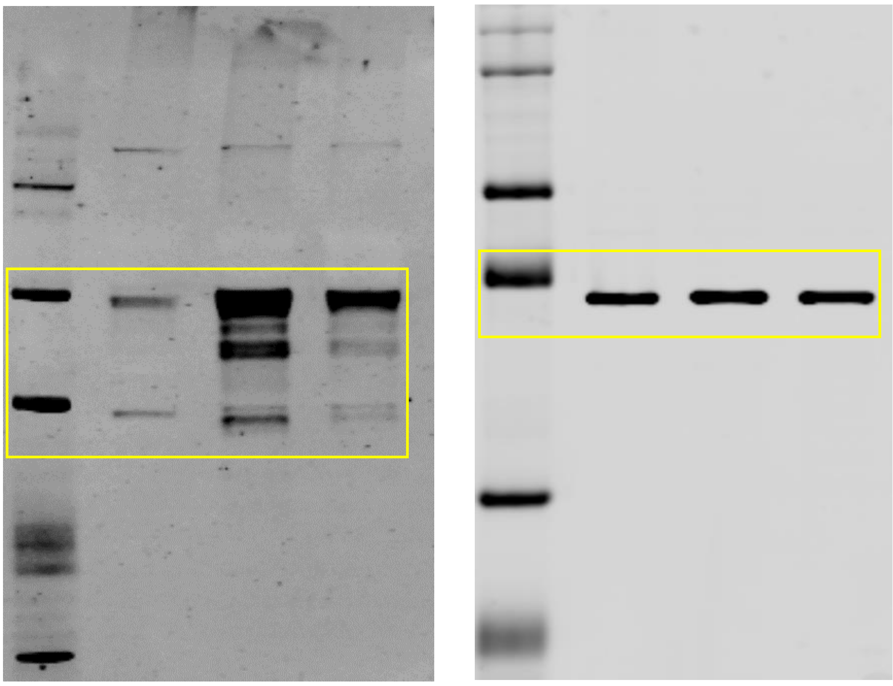

Bottom image (ZR75-1):

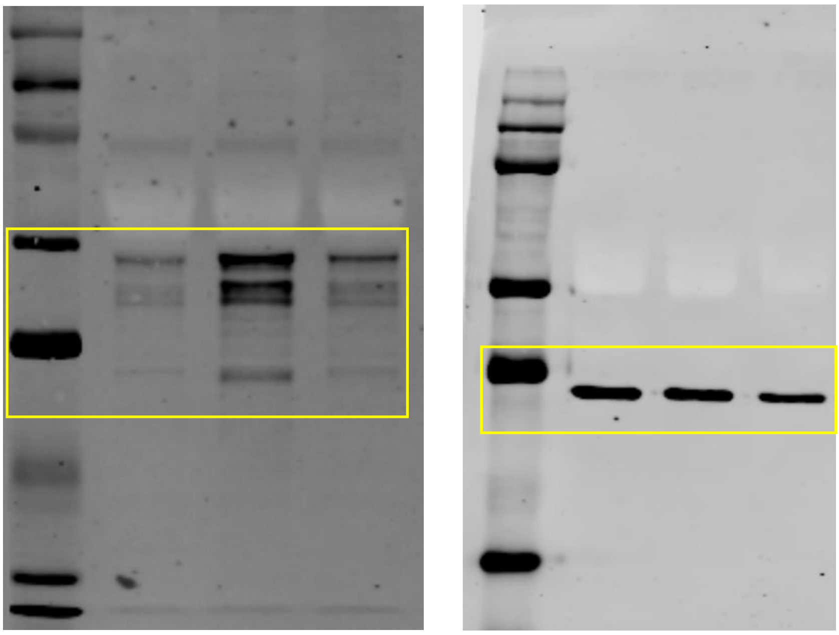

Figure 4A

Left image (MCF-7-LeGO and Δ40p53):

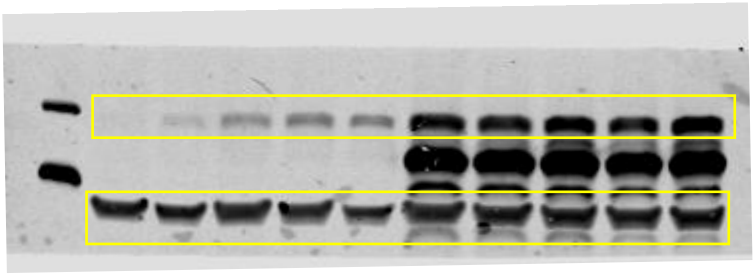

Central image (MCF-7-shNT and shΔ40p53):

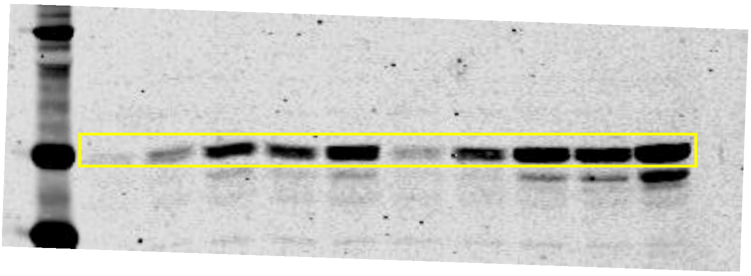

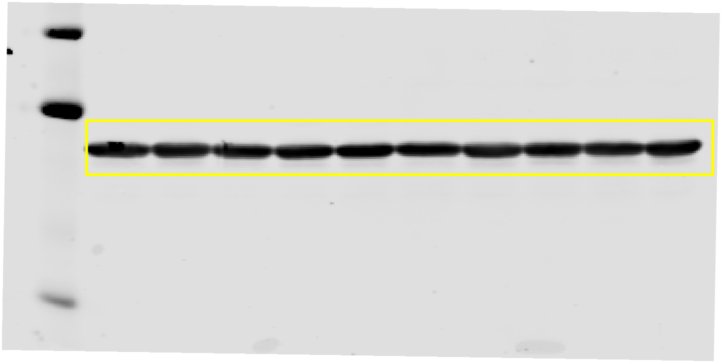

Right image (MCF-7-shp53α):

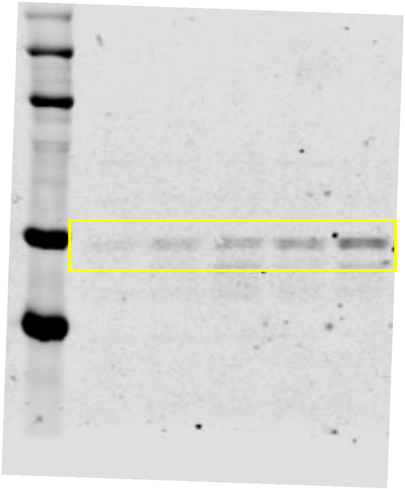

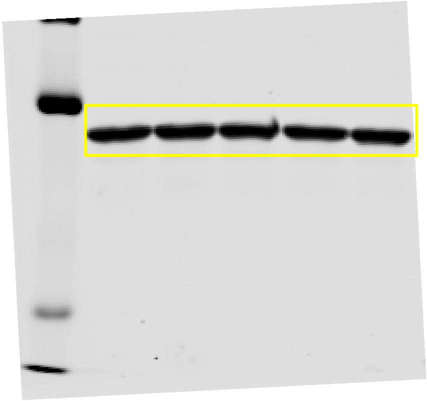

Figure 4E

Top image (MCF-7-LeGO):

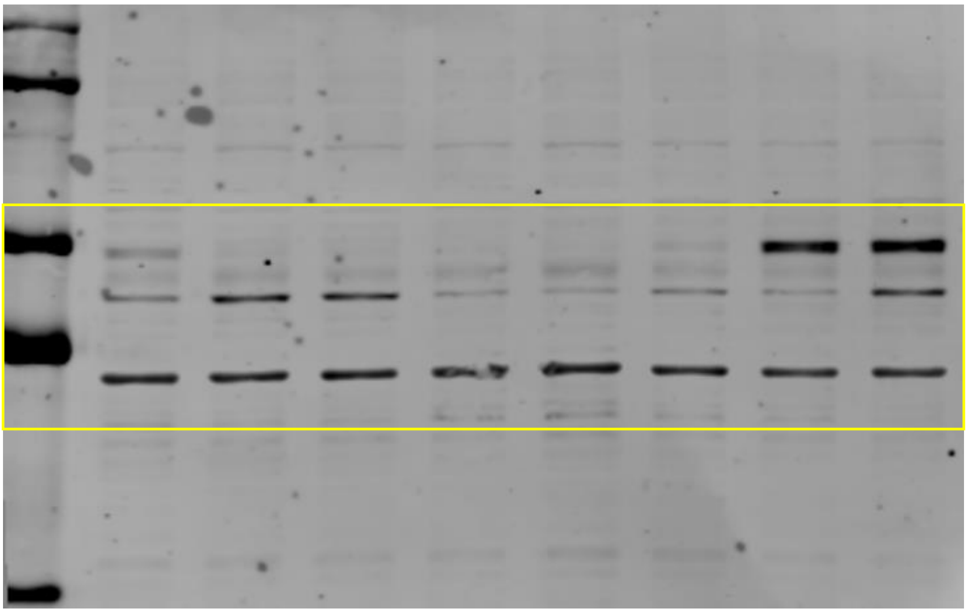

Bottom image (MCF-7-Δ40p53):

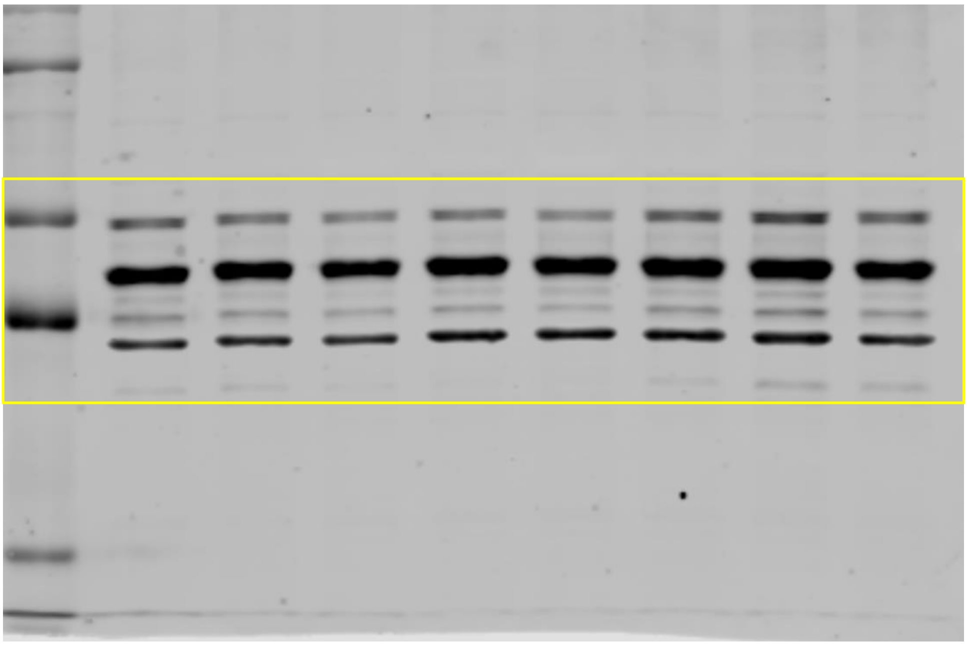

Figure 5D (full blot in Supplementary Figure 3)

Top image (KJC40 antibody):

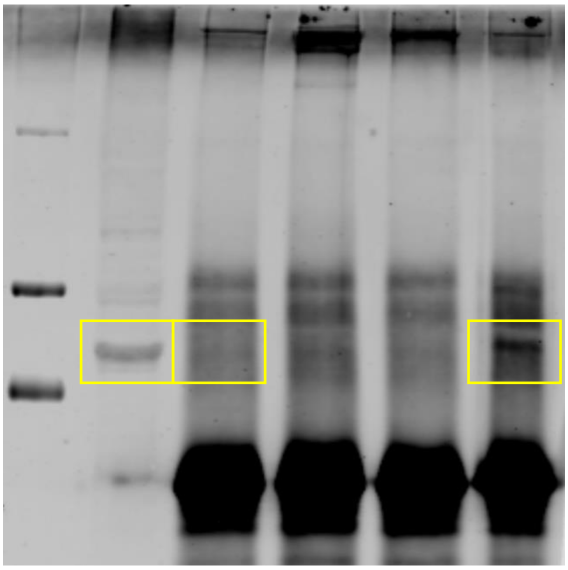

Bottom image (7F5 antibody):

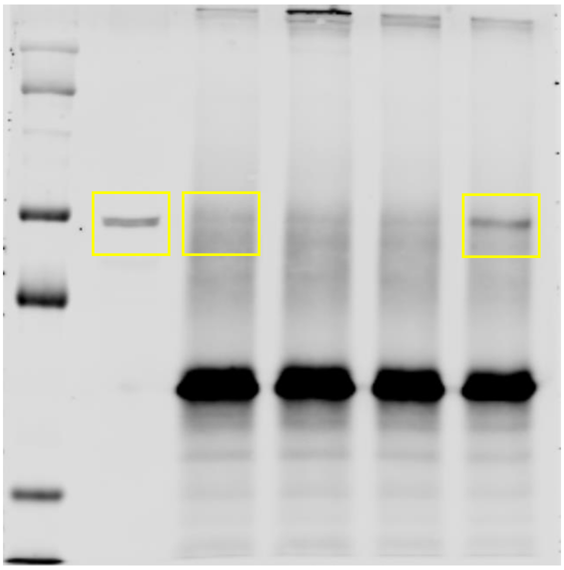

Supplement: Supplementary file 9 — Original Data File [file 41419_2022_5349_MOESM9_ESM.pdf]
